# Next-generation CRISPR systems and advanced delivery paradigms for precision crop improvement: a comparative case study in sugar beet (*Beta vulgaris* L.)

**DOI:** 10.3389/fpls.2026.1900057

**Published:** 2026-09-21

**Authors:** Zulfiya Kachiyeva, Noman Nazik, Muniba Shahid, Rabiah Ashraf, Jawad Ul Hassan, Arshia Amin Khan, Muhammad Huzefa Khan, Huma Mushtaq, Faisal Saeed, Usman Arif, Nurlybek Karimov, Aibek Karimov, Dilyara Gritsenko, Allah Bakhsh

**Affiliations:** 1 Asfendiyarov Kazakh National Medical University, Almaty, Kazakhstan; 2 Seed Biotech Lab, Centre of Excellence in Molecular Biology (CEMB), University of the Punjab, Lahore, Punjab, Pakistan; 3 Laboratory of Molecular Biology, Institute of Plant Biology and Biotechnology, Almaty, Kazakhstan; 4 Nazarbayev University, Astana, Kazakhstan; 5 Research Center AgriBioTech, Almaty, Kazakhstan; 6 Department of Molecular Biology and Genetics, Al Farabi Kazakh National University, Almaty, Kazakhstan

**Keywords:** *Beta vulgaris*, sugar beet, CRISPR/Cas, transgene-free genome editing, genome writing, tissue culture recalcitrance, new plant breeding technologies

## Abstract

Novel, climate-resilient, high-yielding crop varieties are urgently needed to address food security concerns for the world’s growing population. Genome editing techniques have enabled the development of new crop varieties more cheaply, easily, and quickly than conventional breeding methods. A key concern with transgenic crops is producing transgene-free varieties. Transgene-free crops can be developed using new plant breeding technologies and can play a pivotal role in sustainable agriculture. Several techniques can bypass traditional methods for producing edited plants by using hypercompact nucleases and Leaper technology. This review describes various techniques for producing transgene-free crops using combinations of synthetic biology and advanced genome editing tools, such as genome writers, which insert large DNA payloads, and their roles in improving crop genetics. Further, we discuss how these cutting-edge CRISPR techniques can enable the controlled removal of transgenes. The established protocols for tissue culture remain a major hurdle to producing genome-edited plants. The review also outlines several methods that can be used to bypass this laborious tissue culture step, using the recalcitrant crop sugar beet (*Beta vulgaris* L.) (~30% of global sugar production), as a case study to classify each strategy as either already demonstrated in this species or still only proposed by analogy to model- and staple-crop work. In this review, we emphasize how these new plant breeding technologies - and their successful applications in model species- serve as a comparative blueprint to speed up the development of climate-smart sugar beet varieties.

## 1 Introduction

Mutation breeding was coined in 1944 as a new term or technique that opened new horizons for non-specific genome editing (GE) (Martínez-Fortún et al., 2017). Before that, in 1936, the first variety of tobacco was released to the market by inducing mutations (Ran et al., 2017). However, there was a dire need for targeted genome editing, and ultimately the discovery of restriction enzymes gave clear hope for engineering different genomes and making recombinant DNA (rDNA). Later, meganucleases, efficient enzymes naturally found in yeasts and Chlamydomonas (Epinat et al., 2003), became a focus in the late 1970s (Bos et al., 1978; Faye et al., 1979), where they were used to insert introns into the genome. These meganucleases recognize cleavage sites and create a double-stranded break (DSB) by targeting long sequences. This targeted genome editing was first successfully achieved in Tobacco (Zheng et al., 2020). In 1996, a new era of genome editing began with the technique called Zinc Finger Nucleases (ZFNs). ZFNs are a combination of eukaryotic transcription factors (TFs) and bacterial FokI endonucleases that can produce a DSB in DNA. They proved better than meganucleases because they do not require a cleavage site for their action. To date, many plants have been edited using ZFNs including model plant *Arabidopsis thaliana, Nicotiana tabacum, Zea mays, Petunia* spp., *Glycine max, Brassica napus, Oryza sativa, Malus domestica* and *Ficus carica* (Martínez-Fortún et al., 2017; Ran et al., 2017).The use of ZFNs became limited due to laborious design and off-target effects (Kumar et al., 2026). In 2009, a new technique known as Transcription Activator-like Effector Nucleases (TALENs) was introduced. TALENs specifically target the genome and act upstream of the transcription initiation site (Jankele and Svoboda, 2014). In agriculture, the first crop edited using TALENs was rice, engineered to be resistant to blight disease (Demorest et al., 2016; Kelliher et al., 2017).

Besides the efficacy of TALENs, a major issue was their large size, which made delivery and expression in host cells difficult. In addition, TALENs’ repetitive nature also made them incompatible with some viral vectors for delivery (Iqbal et al., 2020). The second generation of genetic engineering technology was adapted for genome editing in 2013, known as clustered regularly interspaced short palindromic repeats (CRISPR) (Gostimskaya, 2022). Among all the GE technologies, the natural and engineered variants of Cas nucleases (Cas9, Cas12a, Cas13) have demonstrated the potential for highly specific genome targeting (Doudna and Charpentier, 2014). A major advantage of CRISPR is that designing guide RNA is straightforward and time-saving (Manghwar et al., 2020). To date, CRISPR has revolutionized the agricultural sector by editing a large number of food and feed crops (Gan and Ling, 2022). Prime editing and base editing are the latest extensions of CRISPR that use nickase Cas9 (nCas9) to produce single-stranded breaks (SSBs), also called nicks, in the target genome and are considered advanced-generation genome editing (Abdullah et al., 2020). This ongoing evolution marks a clear paradigm shift from the generation of simple single-nucleotide polymorphisms and minor indels toward the programming of complex, large-scale structural variations, such as inversions, duplications, and translocations. Consequently, precision DNA insertion technologies are rapidly replacing traditional, random integration methods to meet the advanced requirements of modern plant biotechnology (Gilbertson et al., 2025; Muzaffar et al., 2026).

Currently, CRISPR systems can be divided into two categories. The first involves inserting CRISPR elements into the host genome, followed by screening offspring that do not carry exogenous DNA, vector backbones, or any components such as Cas proteins in the next generation of plants. This category is called transgene-free genome editing (Cheng and Sun, 2025). The other category uses Ribonucleoprotein (RNP) or RNA to induce genome editing, which can be carried out in subsequent generations without integrating foreign DNA. This is known as DNA-free genome editing (Najafi et al., 2023). There are several strategies for screening transgene-free genome-edited plants, such as pigment, fluorescence, and resistance markers, as well as Transgenic Killer CRISPR systems (Cai et al., 2025).

There are still concerns about foreign genes in the host genome, such as Cas9, which is derived from bacteria and can raise immunity concerns. Therefore, safer methods of genome editing are needed, such as the development of LEAPER (Leveraging Endogenous ADAR for Programmable Editing of RNA) technology, which targets RNA rather than integrating into host DNA (Qu et al., 2019a). In this review, we discuss next-generation genome-editing techniques that are useful for producing transgene-free, genome-edited crops without incorporating foreign DNA. The invention of these methods has reduced costs, time, and labor and has helped in the efficient production of transgene-free, gene-edited plants. Remarkable progress has been achieved in DNA-free genome editing crops, including transient transformation methods using protoplasts, viruses, particle bombardment, and Agrobacterium. Besides TKC, there is still a need to use and optimize these technologies in plants. These new plant breeding technologies have revolutionized the scientific boundaries, and in the future, they will help produce sustainable, high-yielding crops resistant to biotic and abiotic stress, compared with previously used conventional breeding and selection methods that require decades to develop a new cultivar.

Sugar beet (*Beta vulgaris* L.) accounts for nearly 30% of global sugar production, making it an economically critical crop. However, its biennial lifecycle and high heterozygosity severely slow down traditional breeding (Eini and Varrelmann, 2022; Misra et al., 2024). Therefore, adapting next-generation genome editing tools to sugar beet is urgently needed. While much of the foundational CRISPR work discussed herein has been established in model and staple crops, these advancements serve as an essential comparative blueprint. Because sugar beet remains poorly represented in this literature relative to staple crops, we treat it throughout as a working test case: for every technology covered, we note whether it has already been demonstrated in sugar beet itself or is so far validated only in model and staple species and merely proposed for translation. By translating these diverse methodologies, researchers can overcome specific bottlenecks in sugar beet, such as its inherent recalcitrance to tissue culture and its susceptibility to devastating viral pathogens.

## 2 Precision engineering for biotic and abiotic stress resilience

By 2050, the need for agricultural production will rise dramatically due to the increase in world population. Currently, demand for agricultural products is increasing because biotic and abiotic stresses negatively affect annual yields. Numerous reports have detailed the severe impact of these abiotic and biotic stressors on global crop yields, highlighting the inadequacies of conventional chemical and biological control methods (Palomares-Rius et al., 2017; Borrelli et al., 2018; Francini and Sebastiani, 2019; Yin and Qiu, 2019; Al Murad et al., 2020; Ding et al., 2020; Manghwar et al., 2020; Hoque et al., 2021; Jagadish et al., 2021; Ali et al., 2022; Saharan et al., 2022; Prasann, 2025). The use of traditional breeding approaches, specifically conventional intergeneric crosses and chemical/physical mutagenesis, offers an alternative strategy to manipulate host resistance mechanisms and enhance crop resilience (Ahmad et al., 2021; Mawcha et al., 2022). Nonetheless, these strategies are characterized by significant limitations, including labor intensiveness, sexual compatibility barriers, and non-specific gene transfer (Wanga et al., 2021; Abdul Aziz and Masmoudi, 2025). Genetic engineering in collaboration with biotechnology offers significant advantages over traditional breeding, enabling precise genome editing and the insertion of desired traits into the host genome across species barriers. Despite its success in enhancing plant stress tolerance, transgenic technology faces challenges such as cost, single-gene dependency, target species adaptation, and time-consuming regulatory clearance (Prado et al., 2014; Van Schie and Takken, 2014). Since 2013, genome-editing technologies, particularly CRISPR-Cas systems, have offered a more suitable alternative due to their precision, versatility, cost-effectiveness, and ability to modify multiple genes simultaneously, enabling the creation of non-GM crops exempt from strict regulations (Zhang et al., 2020; Wang et al., 2022; Ahmad et al., 2023).

Abiotic stress, such as drought, heavy metals, temperature fluctuations, and saline-alkali conditions, induces various physiological disruptions in plants, including ionic imbalance, cellular toxicity, osmotic stress, oxidative stress, membrane disorganization, and disruption of metabolic homeostasis (Ismail and Horie, 2017; Shi et al., 2018; Chauhan et al., 2020; Gupta et al., 2020; Tang et al., 2020; Hoque et al., 2021). To address these stress factors, CRISPR/Cas technology is extensively employed to enhance resilience in various crops. For instance, tomato plants with reduced stomatal density were obtained through CRISPR/Cas9-mediated mutagenesis of *SlGT30* (Solanum lycopersicum Trihelix transcription factor 30), resulting in enhanced drought resistance (Lv et al., 2025). Furthermore, a wealth of studies has successfully demonstrated CRISPR/Cas9-mediated knockout of various susceptibility genes to confer tolerance against drought, salinity, heavy metal toxicity, and extreme temperatures across multiple plant species (Tang et al., 2020; Baeg et al., 2021; Zheng et al., 2021; Li et al., 2022; Xu et al., 2022; Zhang et al., 2022; Deng et al., 2024; Nguyen et al., 2025; Pan et al., 2025). In addition, the CRISPR/Cas9 platform has been employed in the japonica rice variety ZH11 to generate mutants with lower levels of Cd and Pb (Chu et al., 2022). 733Srr22-T1447–1 and HZrr22-T1349–3 were two other mutant rice lines with enhanced salt tolerance obtained by using CRISPR/Cas9-mediated knockout of the *OsRR22* gene (Sheng et al., 2023). While *slmapk3* mutants of Solanum lycopersicum obtained using the CRISPR/Cas9 technique showed increased expression of heat shock proteins (HSPs) and heat stress transcription factors (HSFs) and higher antioxidant enzyme activity, thus directly enhancing thermotolerance (Yu et al., 2019). In sugar beet, translating this model-crop progress to abiotic stress is most advanced for salinity. CRISPR/Cas9-mediated editing of the transcription factor *BvbHLH93* lowers reactive oxygen species accumulation and confers salt tolerance, though the effect is salt-specific and does not extend to drought resistance (Wang et al., 2021). For alkaline, cold, and heavy-metal stress, only candidate loci (WRKY-family transcription factors, raffinose/galactinol synthase genes, and the metal transporters *BvHMA3* and *BvNRAMP3)* have been proposed by analogy to model crops with no CRISPR editing yet reported in sugar beet itself.

Biotic stress poses a significant challenge to sustainable crop production. These stresses arise from living organisms, including phytopathogens (fungi, bacteria, viruses) and insect pests, which disrupt plant physiological processes, consume nutrients, and damage tissues. Substantial progress has been achieved using CRISPR/Cas9 knockout technology, leading to the development of crop varieties that are highly resistant to various fungal, bacterial, and viral diseases, as well as insect pests (Chen et al., 2019).

Plants experience a type of biotic stress caused by a non-cellular entity, the virus. To enhance plant resilience to viral attack, the CRISPR/Cas approach has been employed. The extensive phylogeny and devastating agricultural impact of plant viruses, both DNA and RNA-based, necessitate these advanced, targeted cellular strategies (Dolja et al., 2020; Ali and Mahfouz, 2021; Amorim et al., 2022; Kan et al., 2022; Robertson et al., 2022; Wang et al., 2022; Xavier and Whitfield, 2023). Zhan et al. (2023) demonstrated the use of CRISPR/Cas13d to target and cleave the genome of potato viruses (Zhan et al., 2023). In addition, CRISPR/Cas technology has been used to enhance resistance in wheat, tomato, and maize to wheat yellow mosaic virus (WYMV), yellow leaf curl virus, and sugarcane mosaic virus (SCMV), respectively (Ghorbani Faal et al., 2020; Ogata et al., 2020; Kan et al., 2022). In sugar beet specifically, CRISPR/Cas9 has recently been successfully deployed to combat severe viral diseases that threaten global sugar yields. For instance, highly efficient resistance against the Beet curly top Iran virus (BCTIV) was achieved by using CRISPR/Cas9 to directly target and cleave the viral genome, securing plant defense against this devastating becurtovirus (Yıldırım et al., 2023). Furthermore, CRISPR-mediated knockout of the host susceptibility gene *Bv-eIF(iso)4E* successfully conferred recessive resistance against the Beet chlorosis virus (BChV), demonstrating the power of host-factor manipulation in sugar beet (Rollwage et al., 2024).

Nematodes, parasitic plants, fungal mycopathogens, and phytopathogenic bacteria are major biotic stressors that significantly reduce global crop productivity. These challenges underscore the need for precise genetic editing tools such as CRISPR/Cas systems. Extensive research highlights the complex infection mechanisms of these diverse pathogens, alongside a growing list of successful CRISPR-mediated interventions that disrupt host susceptibility genes to provide broad-spectrum resistance (Jeon et al., 2020; Mishra et al., 2021; Pramanik et al., 2021; Kumar et al., 2022; Bui et al., 2023; Dutta et al., 2024; Alkemade et al., 2025; Kaniganti et al., 2025; Kumar, 2025). For instance, knocking out the *MLO* gene in soybean confers resilience to powdery mildews (Bui et al., 2023). Another CRISPR-Cas9-edited mutation in the SlDMR6–1 gene in tomato confers resistance to a wide array of pathogens, including bacteria (Thomazella et al., 2021). Jhu et al. (2022) obtained CRISPR transgenic tomatoes with enhanced resistance to Cuscuta campestris (Jhu et al., 2022). Researchers employed CRISPR-based loss-of-function mutations in *OsHPP04* and *α-SNAP* genes in rice and soybean to bolster resistance against root-knot endoparasites (Huang et al., 2023; Usovsky et al., 2023). In sugar beet, demonstrated biotic-stress editing is currently limited to a single locus: CRISPR/Cas9 knockout of HS4 (an endoplasmic-reticulum rhomboid protease) in the resistant cultivar NEMATA converts it to full susceptibility to the beet cyst nematode (*Heterodera schachtii*), confirming HS4 as the key resistance determinant (Kumar et al., 2021b). Fungal, bacterial, and insect defenses remain at the candidate-gene stage. Proposed fungal targets include the chitinase and major latex protein genes *BvSP2, BvSE2, BvMLP1*, and *BvMLP3*, both upregulated in resistant cultivars during *Cercospora beticola* infection. A proposed bacterial target is the *NPR1* homolog, to prime salicylic-acid-mediated resistance against *Agrobacterium tumefaciens*; and susceptibility-gene precedents from other crops (e.g., *OsSWEET* promoters in rice, *CsLOB1* in citrus) offer a template for future work on vascular pathogens such as *Xanthomonas beticola*, not yet characterized in sugar beet. Insect resistance in sugar beet still relies on transgenic Cry-toxin expression rather than editing (Jafari et al., 2009). Important studies showing resistance to various environmental stresses achieved in different species using genome-editing techniques are listed in **Table 1** below.

**Table 1:** Representative CRISPR/Cas9 editing strategies for improving abiotic stress tolerance and biotic resistance in plants.

Stress	Plant species	Gene of interest	CRISPR Vector	sgRNA promoter	Transformation method	Reference
Drought	*Oryza sativa*	*OsERA1*	pRGEB31	*OsU3*	*Agrobacterium tumefaciens-*mediated transformation: strain LBA4404	(Ogata et al., 2020)
	*Elymus nutans*	*EnTCP4*	pCAMBIA1300	*OsU6*	*A. tumefaciens-*mediated transformation: strain LBA4404	(Li et al., 2025)
	*Glycine max*	*GmHdz4*	pBGK041	*GmU6*	*A. tumefaciens-*mediated transformation: strain K599	(Zhong et al., 2022)
	*Oryza sativa*	*OsDST*	p2XSgR	*OsU3*	*A. tumefaciens-*mediated transformation: strain EHA105	(Santosh Kumar et al., 2020)
Heavy metals	*Oryza sativa*	*OsNRAMP1*	pYLCRISPR	*OsU3, OsU6a*	*A. tumefaciens-*mediated mature embryo derived calli transformation: strain EHA105	(Chu et al., 2022)
	*Arabidopsis thaliana*	*OXP1*	pHEE401E	*U6-26P, U6-29P*	*A. tumefaciens-*mediated floral dip method: strain GV3101	(Baeg et al., 2021)
Salinity	*Triticum aestivum*	*TaHAG1*	pWMB123-Cas9	*Ubi*	*Agrobacterium*-mediated transformation of immature embryos	(Zheng et al., 2021)
	*Solanum lycopersicum*	*SlHyPRP1*	pCAMBIA	*Ubi*	*A. tumefaciens -*mediated transformation	(Tran et al., 2021)
	*Arabidopsis thaliana*	*AITR3, AITR4*	pHEE401E	*U6-26, U6-29*	*A. tumefaciens-*mediated floral dip transformation	(Chen et al., 2021)
	*Oryza sativa*	*OsRR22*	pYLCRISPR/Cas9	*U3, U6a*	*A. tumefaciens-*mediated transformation	(Sheng et al., 2023)
	*Zea mays*	*ZmIAA9, ZmbHLH32*	pCAMBIA	*U6*	*A. tumefaciens-*mediated transformation of embryos	(Yan et al., 2023)
Temperature	*Oryza sativa*	*OsMYB30*	pYLCRISPR/Cas9	*U6a*	*A. tumefaciens-*mediated transformation	(Zeng et al., 2020)
	*Oryza sativa*	*OsVPE2*	pCAMBIA1300	*Ubi*	*A. tumefaciens-*mediated transformation: strain *EHA105*	(Deng et al., 2024)
Bacterial Disease	*Oryza sativa*	*OsNRAMP1*	pU1301	*U3, U6*	*A. tumefaciens-*mediated transformation of calli	(Chu et al., 2022)
	*Solanum lycopersicum*,	*SlJAZ2*	pK7-CAS9	*Ubi*	*Agrobacterium-*mediated transformation of cotyledons	(Ortigosa et al., 2019)
Viral disease	*Solanum tuberosum*	*Virus Coat protein*	pPTG	*U6*	*Agrobacterium-*mediated transformation: strain GV3101	(Zhan et al., 2023)
	*Solanum lycopersicum*	*Virus Coat protein*	prgsCaM	*AtU6*	*Agrobacterium-*mediated transformation	(Ghorbani Faal et al., 2020)
	*Oryza sativa*	*OsERA1*	pCAMBIA1300	*Not specified*	*A. tumefaciens* -mediated transformation of immature embryos	(Ogata et al., 2020)
	*Beta vulgaris*	*Viral genome (BCTIV)*	pYLCRISPR/Cas9Pubi-H	*AtU6*	*A. tumefaciens* -mediated transformation	(Yıldırım et al., 2023)
	*Beta vulgaris*	*Bv-eIF(iso)4E*	pKSE401	*AtU6*	*A. tumefaciens* -mediated transformation	(Rollwage et al., 2024)
Fungal disease	*Solanum lycopersicum*	*SlPMR4*	pAGM4723	*AtU6*	*A. tumefaciens-*mediated cotyledons transformation: strain AGL1	(Santillán Martínez et al., 2020)
	*Vitis vinifera*	*VvMLO*	pYLCRISPR	*AtU3b, AtU6-1*	*A. tumefaciens-*mediated transformation: strain: GV3101	(Wan et al., 2020)
Insects	*Glycine max*	*GmCDPK38*	pSC1-CRISPR/Cas9	*U6*	*A. tumefaciens-*mediated cotyledon-node transformation method: strain EHA105	(Li et al., 2022)
		*GmUGT*	pSC1-CRISPR/Cas9	*GmU6*	*A. tumefaciens-*mediated transformation: strain EHA105	(Zhang et al., 2022)
	*Arabidopsis thaliana*	*GmUGT*	pFGC5941	*Not applicable*	*A. tumefaciens* floral dip method transformation: strain EHA105	(Zhang et al., 2022)

## 3 Improvement of crops through genetic engineering

Improved crops with higher yields are a major source of global food security. Several tools and techniques have been developed over the years to provide living things with sustainable food and feed resources. The past century was marked by predominantly conventional approaches to sustainability and crop improvement until the development of genome editing techniques in the last two decades (Tiwari et al., 2021). One of the foremost was meganucleases, which adeptly recognize up to 30 base pairs to create homologous double-stranded DNA breaks for efficient targeted modifications in the genome. Subsequently, Zinc Finger Nucleases (ZFNs) and Transcription Activator-like Effector Nucleases (TALENs) were leveraged to generate pathogen-resistant, improved traits and high-yielding crops (Guo et al., 2023). In sugar beet, these delivery routes remain far less tractable. Leaf-protoplast transfection is achievable, but regenerating whole plants from protoplasts is highly unreliable, so stable Agrobacterium-mediated transformation of hypocotyl-derived callus remains the standard route to whole-plant edits, which was validated by CRISPR/Cas9 knockouts of the visual marker genes magnesium chelatase (*BvMgCh*, 60.0% editing efficiency) and phytoene desaturase (*BvPDS*, 68.75% editing efficiency) (Khan et al., 2026). Selection marker choice is also critical as hygromycin selection yields 1.5–5.1% stable transformation, while kanamycin, phosphinothricin, and glyphosate selection perform markedly worse (Miroshnichenko et al., 2026).

These early genome editing techniques had major practical and technical drawbacks in design, delivery, and engineering approaches. CRISPR/Cas refined genome editing technology with its efficiency, adaptability, and expanding novel, precise gene editing. CRISPR/Cas technology has transformed the agricultural field in trait development, domestication, and precise breeding, extending its capabilities to improve crop yield, nutritional content, and sustainability (Kumar et al., 2021a; Dongariyal et al., 2023). In parallel, CRISPR is continually applied to generate insect/pest-resistant, abiotic- and biotic-stress-resistant, and biofortified, higher-yielding crops. The emergence of CRISPR enables the scientific community to knock out susceptibility genes that help pathogens survive against host immunity (Kumar et al., 2022).

Moreover, multiplex genome editing of polyploid crops enables researchers to target and develop valuable complex-trait crops. For instance, multiplex genome editing of a wild tomato transformed it into a more productive crop, expressing 500% more lycopene than the wild type. CRISPR/Cas9 technology played a major role in creating Japan’s first genetically engineered tomato variety, supplemented with gamma-aminobutyric acid (GABA), crucial for human health (Yadav et al., 2024). Furthermore, numerous studies have successfully utilized CRISPR-Cas9 to enhance critical agronomic traits, such as virus resistance, shelf-life, photoperiodicity, and pod shattering resistance across key staple crops like soybean and potato (Enciso-Rodriguez et al., 2019; Hegde et al., 2021; Kieu et al., 2021; Yao et al., 2023; Bi et al., 2024; Mohamed et al., 2024; Pal et al., 2025).

CRISPR/Cas system applications have advanced the agricultural field by enhancing the nutritional value, texture, and flavor of staple crops to ensure food security (Zulfiqar et al., 2024). Genome editing of the *PK1* gene in wheat (*Triticum aestivum* L.) increased zinc accumulation by lowering phytic acid concentration. Similarly, introducing the OsNRAMP2 gene in rice improved iron utilization and distribution (Robertson et al., 2022). Biofortification, known as adding essential nutrients to plants, offers a long-term solution for people who don’t eat a balanced diet. The iron (Fe) content of rice grains is increased by CRISPR/Cas9-mediated silencing of the *vacuolar iron transporter (VIT)* gene. Additionally, the rice mutant *arsenite-tolerant 1 (astol1)*, which has an enhanced trait, significantly increased the amount of selenium in the grains (Tanin et al., 2024). Additional extensive research has demonstrated CRISPR’s capacity to modulate fatty acid profiles, elevate protein content, improve floral fragrance, and reduce anti-nutritional factors or enzymatic browning across diverse crops including mustard, soybean, and potato (Okuzaki et al., 2018; Wang et al., 2019; Karunarathna et al., 2020; Oleszkiewicz et al., 2021; Song et al., 2022; Tiwari et al., 2022; Basu et al., 2023; Mann et al., 2023).

Cytoplasmic Male Sterility (CMS) underpins commercial sugar beet hybrid seed production, governed by genetic conflict between nuclear restorer-of-fertility *(Rf)* genes and the Owen-type (S-type) mitochondrial genome (Matsuhira et al., 2012). High-resolution mitochondrial assemblies have mapped candidate chimeric ORFs and Rf loci for precision editing, but mitoTALEN-based restoration of male sterility has so far only been demonstrated in staple crops such as rice; delivering editing machinery transgene-free across the mitochondrial double membrane remains unverified in sugar beet itself (Zhong et al., 2025). Until this delivery barrier is resolved, CMS-based hybrid breeding in sugar beet will continue to rely on classical backcrossing and marker-assisted selection rather than direct mitochondrial editing.

New developments in CRISPR technology, especially in base editing and prime editing, further enhance proficiency in crops. For instance, base-editing applications could be used to eliminate raffinose family oligosaccharides (RFOs), an anti-nutritional element in soybean that reduces protein digestibility in feeders (Menelih et al., 2025). Furthermore, these advanced editing techniques can be applied to improve crops in both nutritional and yield-associated characteristics, such as flowering period, seed size, and oil concentration. Several examples of enhanced cultivar traits using genetic modification are enlisted below (**Table 2**).

**Table 2:** Representative examples of genome-edited crops for improved agronomic, quality, and biofortification traits.

Crop	Gene	Gene editing	Traits imposed	Reference	
Rice	*OsFAD2-1A, OsFAD2-1B*	Knockout	High oleic acid proportion	(Abe et al., 2018)	
Rice	*OsNRAMP2*	Loss of Function	Iron mobilization	(Yang et al., 2023)	
Wheat	*TaIPK1*	Loss of Function	zinc accumulation and lowers phytic acid	(Ibrahim et al., 2022)	
Soybean	*FAD2-1A, FAD2-1B*		Improved oil quality	(Jo et al., 2022)	
Soybean	*GmPRR3b*	Loss of function	Early flowering and high growth	(Li et al., 2020)	
Soybean	*GmPRR37*	Loss of function	Development of cultivar suitable to grow at high altitudes	(Wang et al., 2020)	
Sugar Beet	*magnesium chelatase (BvMgCh)*	CRISPR/Cas9 knockout, stable Agrobacterium-mediated hypocotyl callus	Visual marker platform; yellow/variegated phenotype, 60.0% editing efficiency, no growth penalty	(Khan et al., 2026)	
	*phytoene desaturase (BvPDS)*	CRISPR/Cas9 knockout, stable Agrobacterium-mediated hypocotyl callus	Visual marker platform; albino/variegated phenotype, 68.75% editing efficiency	(Khan et al., 2026)	
	*Mitochondrial chimeric ORFs / nuclear Rf loci*	mitoTALENs or mitochondrial base editors — proposed, not yet demonstrated	Targeted restoration/engineering of male sterility for CMS hybrid seed production	(Matsuhira et al., 2012; Zhong et al., 2025)	
	*BvSUT4 / BvFL1*	CRISPR/Cas9 or prime-edited promoter — proposed, not yet demonstrated	Suppresses vacuolar sucrose efflux; reduces premature bolting and post-harvest sucrose loss	(Pfeiffer et al., 2014)	
Soybean	*GmSPLs*	Loss of function	Change in plant architecture, increased node and branch number	(Bao et al., 2019)	
Tomato	*Pectate lyase, Polygalacturonase 2a & β-galactanase*	Loss of function	Longer fruit shelf life	(Wang et al., 2019)	
Tomato	*SlALC*	Gene replacement	Longer fruit shelf life	(Gao et al., 2024)	
Tomato	*SP, SP5G, SlCLV3, SlWUS, & SlGGP1*	cis-regulatory alleles	Increase fruit production, ascorbic acid synthesis and salt resistance	(Li et al., 2018)	
Tomato	*Multiple genes*	cis-regulatory alleles	Developed cultivars suitable for urban farming	(Li et al., 2020)	
Potato	*StPPO*	Loss of function	Reduction in enzymatic browning of tubers		(González et al., 2020)

## 4 Strategies to make transgene-free plants

Conventional gene-editing methods introduced DNA constructs into plants via particle bombardment or Agrobacterium tumefaciens, and the resulting plants were identified using a selection marker gene (Gu et al., 2021). Genome-edited plants were distinguished from transgenic plants by sequencing, yielding transgene-free plants. Further crossing is required, which makes the process laborious and impractical for some plants. While sexual segregation effectively removes transgenic footprints in seed-propagated annual model systems, this approach is highly impractical for polyploids, vegetatively propagated crops, and species with complex reproductive barriers (He and Zhao, 2020; Bhattacharjee et al., 2023). In sugar beet, conventional genetic segregation is severely restricted by its biennial vernalization cycle, which requires a 1-to-2-year cold treatment to set seed, and its high heterozygosity and complete self-incompatibility, which trigger severe inbreeding depression and disrupt elite parental genotypes (Gaoua and Arslan, 2026; Miroshnichenko et al., 2026). To overcome these challenges, several strategies are discussed here.

### 4.1 The transgene killer CRISPR

Among the technologies for producing transgene-free plants, one technique is Transgene Killer CRISPR. During tissue culture and regeneration, guide RNAs and Cas9 are expressed to modify target genes and produce genome-edited crops. Transgene Killer CRISPR (TKC) produces transgene-free plants containing suicide genes that target Cas9 and gRNA, thereby eliminating transgene-bearing gametes and embryos in the T_0_ generation (He et al., 2018). The expression of *Barnase* and *CMS2*, two suicidal genes in the CRISPR sequence, is lethal to plant cells owing to their nuclease activity and mitochondrial disruption, respectively. Both suicidal genes are controlled by specific promoters, including *REG2* and *35S*, to ensure their expression at the early embryonic stage. During T_0_ growth, the expression and activation of *Barnase* and *CMS2* eliminate the transgene from pollen and embryos of genome-edited plants, facilitating the germination of only transgene-free seeds. The TKC system enables the production of transgene-free rice while maintaining genetic editing efficacy (Yang et al., 2022). Furthermore, TKC supports multiplex gene editing, which can result in various zygosity in T_0_ plants, as it can edit multiple targets with varying levels of efficacy. Without TKC, traces of CRISPR may be restored to an active state in subsequent generations, complicating the analysis of genotypes. TKC enables predictable modification of heritable alleles in T1 plants in Mendelian proportions (Yang et al., 2022).

Many developments have taken place since the initial TKC report. Endogenous promoters, such as the *UBIQTIN* gene promoters, have been replaced with the CaMV 35S promoter, which may have numerous applications across genetic profiles and could affect the expression of suicide genes (He et al., 2022). A single T-DNA insertion increased the proportion of transgene-free seeds by more than twice (25 to 50 percent) by using another pollen-killer gene (*ZmAA1*) in place of the *CMS2* promoter. Researchers have employed fluorescent markers with TKC to address escape cases. They enhanced the selection of transgene-free progeny by adding *DsRed* fluorescence to *ZmAA1*, allowing rapid visualization of transgenic pollen (Yu et al., 2021). Recently, the FMPKC system was used to couple transgenic *DsRed2* with pollen-killer genes, including *Amy1A* or *orfH79*, to accelerate screening and increase the proportion of seeds with zero transgene (Yu et al., 2024).

The latest TKC2 system, coupling RUBY with Cas9 and gRNA, strongly enhances the efficiency of attaining transgene-free plants through its color-tracking capability. RUBY CRISPR units are then joined with some TKC variants to make four new TKC2 systems that remove remaining transgenes in progeny. In contrast to TKC1, RUBY TKC2 is easy to use and environmentally friendly, thriving in numerous crops with suppressed pollen discharge (Zhu et al., 2025). For biennial, outcrossing species like sugar beet, researchers have proposed adapting the TKC framework by coupling *Cre/loxP* or *FLP/FRT* recombinase loops with chemical-inducible or taproot-specific promoters to trigger programmed auto-excision of the Cas9-sgRNA cassette directly in somatic tissues (He and Zhao, 2020; Gaoua and Arslan, 2026). Despite being inapplicable in crops with a high rate of asexual reproduction and in those with gametophytic-killer genes, TKC remains one of the most efficient ways to obtain transgene-free gene-edited plants in species reproducing by seeds.

### 4.2 Nanoparticles based technique as an alternative

Nanoparticles (NPs) have shown promise for developing transgene-free genome-edited plants by delivering CRISPR/Cas reagents to plant cells without introducing foreign DNA. A notable example is pollen magnetofection, which combines positively charged magnetic particles with DNA’s negative charge to form an MNP-DNA complex. After mixing the complex with pollen, a magnetic field is applied, facilitating DNA delivery into the pollen before germination. This method preserves pollen structure and function and yields insect-resistant transgene-free seeds (Zhao et al., 2017). However, the reproducibility of pollen magnetofection remains highly controversial across diverse plant species, with critical evaluations reporting a complete lack of heritable transformation or genomic editing events due to severe physical barriers in the pollen wall (Vejlupkova et al., 2020). In sugar beet, this nanotechnology remains a strictly proposed, unverified strategy, as delivering bulky CRISPR-Cas9 RNPs across the narrow germinal apertures of its exceptionally small pollen grains (18–22 μm in diameter) without triggering cell lysis or loss of viability represents an unsolved biophysical bottleneck (Vejlupkova et al., 2020; Gaoua and Arslan, 2026).

Nanoparticles can also be applied to meristem cells, and genome editing of these cells results in mosaic modifications from which gene-free plants can be obtained. Modified transgene-free plants can also be regenerated in tissue culture or vegetative culture (Gogoi et al., 2025). Carbon nanotubes, silica nanoparticles, metal oxide nanoparticles, and quantum dots are among the nanoparticles that can also be directly adsorbed by plants. Gold nanoparticles (AuNPs), on the other hand, are highly stable and biocompatible. Lipid nanoparticles and gold-based nanomaterials have been shown to safely deliver CRISPR/Cas9 systems (Chen et al., 2019).

One of the most interesting and easiest-to-use approaches is the use of carbon-dot nanoparticles whose surface is covered with plasmid DNA. The method yielded effective Cas9 and gRNA expression and effective genome editing, representing a fast, less expensive, scalable technology that can be extended to numerous crop species. Although they have potential, there are still several problems that should be addressed, such as nanoparticle-cargo binding, compliance with environmental and biosafety regulations, and the establishment of the optimal nanoparticle dose and coating. More research is also needed to determine the long-term consequences of the use of nanoparticles in the editing of plant genes (Doyle et al., 2019). Systematic reviews on nanomaterial-mediated delivery indicate that the vast majority of current plant nanotechnology literature supports transient target perturbation, such as RNAi-mediated gene silencing, rather than the generation of stable, heritable edits in elite crop germplasm. While transient leaf-level assays provide an excellent platform for trait discovery and rapid validation of candidate susceptibility genes (e.g., *MLO* homologs), achieving stable germline transformation remains a major scientific hurdle (Singh et al., 2026).

Despite their potential, routine adoption is hindered by the need for precise nanoparticle-cargo dose optimization to ensure efficient uptake without inducing cytotoxicity. Furthermore, the long-term environmental biosafety of using nanomaterials in open fields remains a critical area for investigation before large-scale commercial use can be realized. Despite these hurdles, delivering substantial DNA constructs across rigid plant cell walls persists as a core bottleneck in the field. This challenge continues to drive intense interest in transient, non-integrating delivery media, such as these advanced nanomaterials and engineered viral vectors, to completely bypass laborious tissue-culture processes (Muzaffar et al., 2026).

In the genus *Beta*, there are currently no verified reports of successful, heritable genome editing using any nanoparticle-based platform, leaving these technologies strictly at the proposed stage (Gaoua and Arslan, 2026; Singh et al., 2026). While target-specific RNA interference (RNAi) delivered via clay nanosheets (BioClay) has been actively proposed as an alternative non-transgenic biocontrol method to protect sugar beet foliage against fungal pathogens without genomic integration, direct RNP translocation across waxy cuticular barriers remains highly restricted. To bridge this complexity gap, researchers have proposed employing polymer-functionalized single-walled carbon nanotubes (SWCNTs) or modified carbon dots to deliver transient editing reagents into the shoot apical meristem of germinating seeds (Demirer et al., 2021). This proposed pathway aims to exploit the passive endosomal transport of hypercompact nucleases across the rigid cell wall size-exclusion limit (~5–20 nm) to regenerate plants without undergoing a laborious tissue-culture phase (Cunningham et al., 2018).

### 4.3 RNP-mediated genome editing

RNP-mediated genome editing has gained recognition as a valuable technique for producing transgene-free plants. In this approach, the Cas protein and guide RNA are assembled into an RNP, which is then delivered into plant cells, as shown in **Figure 1**. Because the gRNA is chemically synthesized, the resulting plants are transgene-free. RNPs remain transiently in plant cells and are then enzymatically degraded, avoiding side effects caused by prolonged genome exposure to CRISPR reagents. This technique can be applied to protoplasts, zygotes, and embryos in various fruits and vegetables (Metje-Sprink et al., 2019). The principal limitation of RNPs is their heavy reliance on protoplast transformation, which is a significant regeneration bottleneck. This labor-intensive and genotype-specific process currently limits the scalability of RNP-mediated editing compared to *in planta* or viral-mediated delivery methods. Although PEG-mediated transfection of preassembled RNPs has successfully yielded transgene-free edited plants in crops like potato, carrot, and sweet potato (Andersson et al., 2018; Ramakrishnan et al., 2025; Yarra and Krysan, 2025), isolating and regenerating plants from cell wall-free protoplasts remains highly technically challenging across most agricultural species (Lokya et al., 2025).

**Figure 1:**
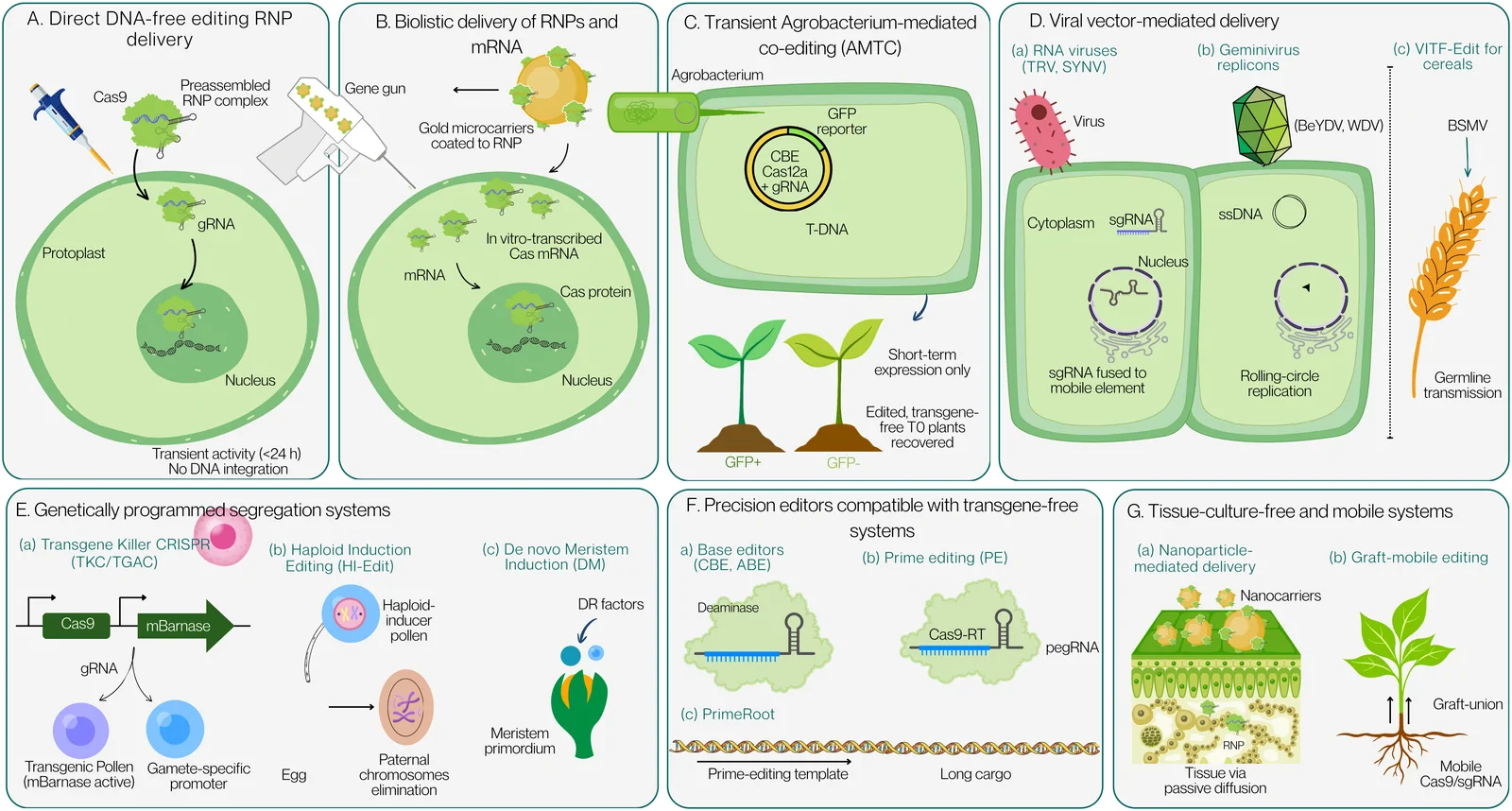
Various approaches to producing transgene-free genome-edited crops. DNA-independent physical delivery **(A, B)**. PEG-mediated transfection or electroporation, or direct intracellular delivery, of pre-assembled Cas9/Cas12a ribonucleoproteins (RNPs) **(A)**, and biolistic delivery of RNPs or optimized *in vitro* transcribed (IVT) mRNA (e.g., v2TMV/DEN2) **(B)**. These techniques limit nuclease activity to a short period (<24 h), thus reducing off-target effects and eliminating genomic integration. **(C)** The non-selected T-DNA expression method of Agrobacterium-mediated transient co-editing (AMTC) is used to produce T0 edits without long-term integration, which are usually screened by fluorescent reporters (such as GFP). **(D)** Viral vectors (RNA viruses (TRV, BSMV), geminivirus replicons (WDV)) are used to express and edit germline cells at high levels without integrating into the host genome. **(E)** Active elimination systems that are genetically programmed to segregate transgenes. This transgene **(E, a)** Transgene Killer CRISPR (TKC) uses a gamete-specific promoter to express a toxin (mBarnase) to ablate transgenic pollen/embryos selectively. **(E, b)** Haploid Induction (HI-Edit) removes paternal chromosomes carrying the CRISPR transgene during zygogenesis. **(E, c)** *De novo* Meristem (DM) induction uses developmental controllers to regenerate edited shoots in the absence of tissue culture. **(F)** Accurate editing sites. Complex trait engineering can be done without double-strand breaks using advanced editors such as Base Editors (BE) to make single-nucleotide transitions **(F, a)**, Prime Editors (PE) to make search-and-replace modifications **(F, b)**, and PrimeRoot to make large-cargo insertions **(F, c)**. **(G)** Genotype-independent mobility. Passive entry into walled plant cells can be achieved by **(G, a)** Nanoparticle-mediated delivery. **(G, b)** Graft-mobile editing uses tRNA-like sequences to deliver Cas9/gRNA transcripts to a non-transgenic scion to produce transgene-free seeds.

In potato, transgene-free plants were produced by delivering preassembled CRISPR/Cas9 RNP into protoplasts to target the GBSS gene. This approach achieved high mutagenesis frequency, and almost all progeny were transgene-free (Andersson et al., 2018). In fruit crops such as grapevine and apple, the technique was applied to protoplasts for precise gene editing. In grapes, the *MLO7* gene was targeted to confer resistance to powdery mildew, and in apples, *DIPM-1*, *DIPM-2*, and *DIPM-4* were targeted for resilience to fire blight (Malnoy et al., 2016). In wheat, CRISPR/Cas9 RNP delivered by particle bombardment into undifferentiated cells achieved effective genome editing. No off-target mutations were detected. Mutagenesis efficiency varies across plants; in wheat, it ranged from 1.3% to 4.4%, and in rice, it was about 14-64% (Lokya et al., 2025). Furthermore, because RNPs persist temporarily in plant tissues, their cleavage kinetics are highly sensitive to microenvironmental factors. For instance, applying high-temperature treatments (such as 30 °C or 37 °C) following biolistic or protoplast delivery has been demonstrated to significantly enhance Cas9 nuclease activity in wheat, boosting mutation frequencies while maintaining tissue viability under selection-free conditions (Poddar et al., 2023). Additionally, to streamline these workflows, rapid *in vivo* protoplast assays can be instituted as a standard pre-validation step to select highly effective sgRNAs before initiating laborious and resource-intensive embryo bombardment and regeneration cycles (Poddar et al., 2023; Pandey et al., 2026).

This method is also adaptable to various delivery techniques. Particle bombardment is used to incorporate RNPs (**Figure 1**) into plant cells and has been applied in plants such as rice, maize, and wheat (Svitashev et al., 2016). Nanoparticle delivery is a cutting-edge method for applying RNP into plants that is species-independent and good for material safety (Zhang et al., 2021). Regeneration of complete plants remains a major challenge, but CRISPR/Cas9 RNP is still a good strategy for producing transgene-free plants across various species and for facing fewer regulatory barriers (Liang et al., 2017).

In sugar beet breeding, although standard plasmid-based transformations remain the norm (Khan et al., 2026), RNP-mediated editing represents a highly promising, proposed blueprint to bypass its severe tissue culture and biennial barriers (Rollwage et al., 2024; Gaoua and Arslan, 2026). While PEG-mediated transfection of sugar beet protoplasts is actively proposed to evaluate guide RNA cutting kinetics on endogenous targets in a transient, high-throughput manner, recovering whole, non-chimeric regenerants is currently blocked by single-cell recalcitrance. To bridge this complexity divide, researchers have proposed delivering preassembled RNPs directly into highly regenerative intact tissues (i.e. shoot apical meristem of germinating seedlings or axillary buds of dissected crowns) using *in planta* particle bombardment (iPB-RNP) or micro-robotic needle injections (Quiroz et al., 2025). By bypassing both plasmid integration and the sterile callus phase, these proposed physical delivery networks would enable the rapid, scarless engineering of elite commercial sugar beet lines, providing a streamlined pathway to develop climate-smart varieties that are exempt from stringent GMO regulations (Miroshnichenko et al., 2026).

### 4.4 Transient genome editing using Agrobacterium

Emerging challenges in inserting foreign genes demand innovative strategies, so a shortcut agrobacterial mutagenesis is used. It involves Agrobacterium strains engineered with a site-specific nuclease expression vector that mediates infection of potato stem cells. As a result, regeneration-related gene activation leads to shoot formation from plantlets that contain genetically modified cells. These shoots arise from combining a regeneration-driving gene with the short-term expression of the genome-editing enzyme gene. Potatoes produced by this method do not contain the foreign sequence of the binary vector used in transformation. This method can be applied to economically important plants, such as heterozygous perennial species that are difficult to regenerate from biolistic transformation (Umemoto et al., 2023). Similarly, in vegetatively propagated or highly heterozygous outcrossing crops like potato and banana, where sexual segregation is genetically disruptive to the elite clonal cultivar background, Agrobacterium-mediated transient expression of CRISPR/Cas9 or base editors (such as cytidine base editors targeting the endogenous *ALS* locus) has successfully produced transgene-free, T-DNA-free edited lines in the T_0_ generation without backcrossing (Van Den Broeck et al., 2025). Although this process yields a small number of T_0_ plants, it usually produces more gene-edited lines than RNP methods. This method is inexpensive and has no specific requirements, so it can be easily performed in labs (Pachauri et al., 2025). In transient Agrobacterium transformation, the regenerated T_0_ population usually consists of three groups: plants that have integrated T-DNA, transiently transformed plants, and untransformed plants. It can remain difficult to identify the small percentage of altered plants that have lost their transgenes, though these ratios will change with different transformation conditions. Improved Agrobacterium strains that minimize stable integration and increase transient expression of CRISPR reagents would enhance the method (He and Zhao, 2020). A good example of this technique is the use of Agrobacterium to express Cas9 and gRNA targeting the *PDS* gene in tobacco. More importantly, in the absence of herbicide or antibiotic selection, the survival of transiently altered cells was possible. Because approximately 10 percent of regenerated plants were modified and free of transgenes, no segregation would be required in the next generation (Coolhaas, 1952). This efficiency can be further optimized; for example, combining a 3-day short-term antibiotic selection with a transient heat treatment (37 °C for 24 hours) on the second day after co-cultivation has been demonstrated to significantly enhance transient expression-mediated editing, facilitating the recovery of transgene-free, *Pds*-edited plantlets directly from tobacco leaf tissues (Kobayashi, 2025). Although Agrobacterium is widely used in numerous plant species, it is a pathogen that causes plant disease, posing management problems in particular regions. Successful genome editing is heavily dependent on an effective delivery method, and one way to achieve it is the temporary expression of CRISPR reagents to produce transgene-free plants with the help of Agrobacterium without the necessity of sexual segregation (**Figure 1**).

In *B. vulgaris*, although stable *Agrobacterium*-mediated hypocotyl callus transformation has recently been established as a reliable editing platform, transient Agrobacterium-mediated delivery remains a proposed strategy to bypass its severe recalcitrance and 2-year biennial cycle. To establish transgene-free, selection-free lines, researchers have proposed executing transient agroinfiltration of Cas9-sgRNA plasmids targeting physiological loci directly into highly regenerative intact tissues, such as blade-petiole transition zones or shoot apical meristems, and applying transient heat stress to maximize nuclease cutting while avoiding stable T-DNA integration (Poddar et al., 2023; Gaoua and Arslan, 2026).

DNA-free approaches present a clear trade-off between high transient precision (RNPs) and genotype-independent scalability (Nanoparticles). However, these technologies offer a major regulatory advantage: in jurisdictions utilizing product-based regulations (e.g., the United States and Canada), crops lacking foreign DNA integration may bypass the stringent, costly evaluations applied to traditional GMOs. This regulatory ‘fast-track’ positions DNA-free delivery as a critical commercial accelerator for rapid crop improvement (Singh et al., 2026).

## 5 Genome editing as a foundation for plant synthetic biology

CRISPR/Cas genome-editing systems have rapidly emerged as a foundational enabling technology in plant synthetic biology, transforming genome manipulation into a programmable, precise, and design-oriented process. In contrast to previous nuclease-based systems, RNA-guided CRISPR/Cas systems enable sequence-specific targeting through easy customization of guide RNA and can be reconfigured for rational targeting of plant genomes at new levels. This programmability has advanced plant genome engineering to multiplex GE, in which multiple loci can be edited simultaneously to rewire intricate biological pathways and regulatory networks. This multiplexing is especially important in the context of synthetic biology, where plant phenotypes and metabolic responses are usually regulated by interactions within gene networks rather than by discrete genetic components (Scheben et al., 2017). Therefore, CRISPR/Cas technologies provide a regulatory platform for building predictable, modular, and scalable genetic designs within plants. CRISPR-based systems for transcriptional regulation, such as CRISPR activation (CRISPRa) and interference (CRISPRi), in addition to genome disruption, have generated new tools for synthetic biology, allowing reversible and tunable control of gene expression without affecting DNA sequence integrity. Dynamic control over endogenous genes is achieved by catalytically inactive Cas proteins (dCas) complexed with transcriptional activators or repressors, enabling fine-tuning of metabolic fluxes, developmental programs, and stress responses (Shrestha et al., 2018; Kovalev et al., 2025). In sugar beet, while dCas9/dCas12a-based transcriptional reprogramming (CRISPRa/i) and synthetic gene circuits are widely recognized as promising tools to engineer environmental resilience, their practical application is still only proposed due to the high heterozygosity and severe tissue culture recalcitrance of the species (Khan et al., 2025; Gaoua and Arslan, 2026). High-resolution contiguous *de novo* genome assemblies like EL10 now offer a predictive *in silico* template to design highly specific multiplex sgRNA arrays and dCas-repressor complexes, aiming to fine-tune vegetative taproot sucrose loading dynamics or pathogen-sensing networks without stable transgene integration (McGrath et al., 2023; Gong et al., 2025). These capabilities support a shift from traditional genetic modification to design-based biology, in which biological systems are constructed with standardized regulatory components, logic-based control, and predictable behavior.

### 5.1 CRISPR-mediated metabolic pathway engineering

Plants are emerging as sustainable biofactories capable of producing a broad range of high-value primary and secondary metabolites, including pharmaceuticals, nutraceuticals, biofuels, and industrial compounds. Nevertheless, native plant metabolic systems have evolved for survival rather than high product yield, resulting in strong flux control, competing branch pathways, and intense feedback on metabolites and physiological parameters. Conventional transgenic techniques have been largely ineffective at overcoming these limitations, in part because of random gene integration, variable expression levels, and the inability to synchronize the activities of multiple pathway components. By comparison, CRISPR/Cas-based GE provides an accurate and scalable system for plant metabolic engineering, enabling the specific modification of numerous genes, regulatory elements, and transcriptional regulators within a pathway (Singha et al., 2022; Wang et al., 2023). This has shifted metabolic engineering from single-gene modification to pathway rewiring, which is critical for achieving predictable and stable metabolic responses.

CRISPR-based metabolic pathway engineering leaves biosynthetic networks open to rational redesign by multiplex editing of rate-limiting enzymes, pathway repressors, and competing metabolic sinks. Simultaneously modulating multiple nodes is often necessary in complex pathways where carbon flux must be redirected to increase the accumulation of metabolites, e.g., in terpenoid, alkaloid, and lipid biosynthesis. RNA-guided nucleases enable more selective knockout of negative regulators, more precise control of enzyme activity through promoter or coding-sequence editing, and transcriptional reprogramming via CRISPR activation or interference systems (CRISPRa/i), which reduces pleiotropic impacts often linked to constitutive overexpression (Ameruoso et al., 2022). Notably, the programmability and multiplexing ability of CRISPR/Cas systems enable the construction of pathway-level designs that are stable, heritable, and compatible with transgene-free and tissue culture-free methods. When translating these metabolic engineering principles to sugar beet, researchers face a critical species-specific boundary between verified monogenic edits and proposed multi-genic pathway redesign. Stably demonstrated editing in the genus *Beta* remains confined to single-gene knockouts, such as *BvMgCh* or *BvPDS* to establish visual screening lines (Khan et al., 2026), whereas multiplexed pathway control remains a strictly proposed strategy. To maximize industrial utility, researchers have proposed deploying CRISPRa/i and base editing to selectively downregulate competing vacuolar invertases while simultaneously upregulating the tonoplast sucrose transporter *BvTST2;1*, thereby redirecting primary carbon flux to maximize taproot sucrose accumulation without inducing vegetative growth penalties (Wang and Demirer, 2023; Keller and Neuhaus, 2025).

### 5.2 CRISPR-mediated terpenoid pathway engineering

The most recent developments in CRISPR/Cas-based GE are enabling increasingly specific and multiplexed regulation of plant terpenoid biosynthesis, which has traditionally been constrained by regulatory bottlenecks that have limited the yields of these high-value specialized metabolites. For example, knockout of genes encoding terpene synthases in *Nicotiana tabacum*, e.g., *NtSPS1*, with CRISPR/Cas9 altered terpenoid profiles, demonstrating that key nodes in the pathway can be modulated to change terpene composition and metabolic bioproducts and to confer resistance to pathogens, e.g., TMV (tobacco mosaic virus), in engineered lines (Dai et al., 2025). An interesting study of CRISPR/Cas-mediated pathway regulation in terpenoid biosynthesis has been conducted in Salvia miltiorrhiza, a medicinal plant with diterpenoid tanshinones. Specific CRISPR/Cas9 editing of an upstream open reading frame (uORF) linked to the SmCPS1 gene, one of the major diterpene synthases, altered post-transcriptional regulation, leading to substantial increases in tanshinone levels without harmful consequences for plant development (Shao et al., 2026). This method exemplifies how regulatory editing, rather than merely gene disruption, can induce flux through specialized metabolic pathways by relieving translational repression of rate-limiting enzymes. Simultaneously, conditional silencing of individual genes in the same species, including *SmCPS4*, has been shown to shift the metabolic pathway toward alternative branches and upregulate a variety of biosynthetic and transcriptional regulators, ultimately leading to a well-coordinated increase in tanshinone content (Fan and Feng, 2025). Beyond model species, CRISPR editing of terpenoid pathways has been successfully extended to crop plants like alfalfa (*Medicago sativa*), where knocking out the saponin biosynthetic genes *CYP93E2* and *CYP72A61* blocked the production of undesirable soyasaponin I, shifting metabolic flux to generate alternative valuable triterpene compounds (Confalonieri et al., 2021). In sugar beet (*Beta vulgaris* L.), triterpene saponins represent a crucial class of specialized metabolites that function as natural chemical defenses but significantly hinder industrial sugar processing by forming stable, problematic foam during aqueous sucrose extraction. Although direct editing of saponin biosynthetic nodes remains a strictly proposed strategy in the genus *Beta*, researchers have proposed utilizing CRISPR/Cas9 or cytidine base editors to selectively knock out candidate oxidosqualene cyclases (such as beta-amyrin synthase) or specific cytochrome P450s (homologs to alfalfa *CYP93E2*) in elite germplasm, thereby eliminating processing-foaming penalties in raw juice without compromising vegetative fitness or cell wall integrity (Confalonieri et al., 2021; Gaoua and Arslan, 2026).

### 5.3 Alkaloid biosynthesis and CRISPR-assisted pathway control

The biosynthesis of alkaloids in plants involves a heterogeneous group of nitrogen-based secondary metabolites that are important pharmaceuticals (e.g., tropane, indole, and benzylisoquinoline alkaloids) and are regulated by a complex network of enzymes and tissue-specific expression patterns. More recent CRISPR/Cas9 interventions have begun to decipher these pathways, allowing specific biosynthetic nodes to be edited with precision and minimal pleiotropic effects. For example, a CRISPR/Cas9 knockout of the PYRROLIDINE KETIDE SYNTHASE (PYKS) in Atropa belladonna hairy roots caused a dramatic decrease in tropane alkaloid accumulation, demonstrating that targeted loss-of-function mutations can be used to elucidate enzyme roles, divert metabolic flux for pathway discovery, or inhibit the production of unwanted metabolites (e.g., hyoscyamine, scopolamine) in medicinal plants (Das et al., 2024). These studies not only highlight the importance of CRISPR in dissecting highly regulated alkaloid networks but also suggest that branch points and regulatory factors underpinning metabolite specificity may be modulated using CRISPR as a basis to recapitulate metabolic rewiring in plant synthetic biology (Hu et al., 2024). However, engineering these complex pathways can introduce critical trade-offs. For instance, multiplex CRISPR/Cas9-mediated knockout of the core nicotine biosynthetic genes *A622* and *BBL* in tobacco (*Nicotiana tabacum*) successfully lowered leaf nicotine levels but induced severe foliar browning and developmental abnormalities, demonstrating the tight evolutionary linkage between nitrogenous secondary metabolism and vegetative growth (Jeong et al., 2024). Translating these synthetic biology paradigms to *B. vulgaris* reveals a distinct taxonomic profile, as the genus *Beta* does not synthesize traditional nitrogenous alkaloids. Upregulating endogenous *choline monooxygenase* (*BvCMO*) and *betaine aldehyde dehydrogenase* (*BvBADH*) genes via inducible dCas9-VP64 transcriptional activators represents a promising proposed strategy to boost glycine betaine accumulation and enhance salt and cold resilience under environmental stress (Tresballes, 2005). In parallel, delivering the betalain-biosynthetic *RUBY* reporter cassette alongside transient CRISPR reagents is proposed as a non-destructive visual selection system to identify edited, transgene-free sugar beet tissue directly on hormone-free media, completely bypassing the physiological stress of chemical selection agents (Zhu et al., 2025).

### 5.4 Lipid and fatty acid biosynthesis editing for quality and yield

Recent advances in CRISPR/Cas-based editing have shown that targeted manipulation of lipid metabolic genes can produce meaningful improvements in oil content and composition in important oilseed crops, aligning with synthetic biology objectives to improve agronomic and nutritional characteristics. CRISPR/Cas9 knockouts of seed fatty acid reducer (SFAR) (*GmSFAR4a/b*) genes in soybean (*Glycine max*) significantly increased total seed oil content, to about 8% under controlled greenhouse conditions and as high as 17% in plants grown in the field, without impairing cell growth or seed viability, thereby providing an example of how editing a small group of lipid metabolism regulators can unlock latent biosynthetic potential (Liao et al., 2025). This precise pathway control has been extended to modulate fatty acid profiles in other oilseed species; for instance, multiplex CRISPR/Cas9 editing targeting the fatty acid desaturase 2 (*BnFAD2*) and fatty acid elongase 1 (*BnFAE1*) homeologs in rapeseed (*Brassica napus*) boosted seed oleic acid content to 70%–80% (Liu et al., 2022). To avoid pleiotropic developmental penalties in vegetative tissues, researchers have also edited the introns or seed-specific regulatory elements of the peanut *FAD2* gene, selectively increasing oleic acid levels strictly within seeds without altering fatty acid compositions in leaves or roots (Neelakandan et al., 2022). These highly selective edits can leverage the modularity of CRISPR systems to tune degradation pathways and channel carbon flux toward improved triacylglycerol production (seed oil), providing an accurate way to use metabolic engineering to complement more traditional breeding and systems biology methodologies. These developments show that CRISPR platforms can not only modify quantitative lipid characteristics but also guide genetic designs that trade off storage lipid biosynthesis against total plant fitness, a fundamental problem in the development of sustainable oilseed crops. In sugar beet, which accumulates sucrose rather than triacylglycerols, manipulating lipid metabolism represents a vital proposed pathway to enhance environmental tolerance. Specifically, remodeling membrane desaturase genes (such as *BvFAD2* or *BvFAD6*) via base or prime editing is proposed as a key strategy to modulate lipid desaturation, stabilizing chloroplast and tonoplast membrane fluidity to prevent cold-induced sweetening or cellular rupture under freezing conditions (Gaoua and Arslan, 2026). Additionally, because sugar beet is highly sensitive to foliar transpiration water loss, editing epicuticular wax biosynthetic genes (such as 3-ketoacyl-CoA synthase (*KCS*) homologs) is proposed to reprogram epicuticular wax structure, providing a robust drought and salt-resilience mechanism across elite breeding lines (Demirer et al., 2021).

### 5.5 CRISPR-based synthetic promoters and programmable regulatory circuits in plant synthetic biology

Accurate regulation is a core part of plant synthetic biology because pathway engineering based on metabolic pathway composition requires not only gene expression dynamics but also precise regulation. Recent CRISPR/Cas technologies have enabled direct editing of endogenous cis-regulatory elements, such as promoters, enhancers, and untranslated regions, allowing transcription to be quantitatively modulated without any change in coding sequences. One of the earliest applications of this method was the editing of promoter regions that regulate yield-related genes in tomato using CRISPR/Cas9, in which minor nucleotide alterations in cis-elements yielded a spectrum of expression values and predictable phenotypic changes without the need to integrate transgenes (Hernandes-Lopes et al., 2023). Beyond simple indels, precise knock-in of transcriptional enhancers represents a major leap in regulatory engineering. For example, Yao et al. (2024) achieved up to an 869-fold enhancement in gene expression by inserting short transcriptional enhancers (STEs) upstream of rice promoters. Similarly, the insertion of a viral-derived translational enhancer (AMVE) into the 5’ untranslated region (5’ UTR) boosted endogenous protein expression up to 2.8-fold in rice, conferring robust salinity tolerance (Shen et al., 2023). Precise promoter-level knock-ins have also been harnessed to insert pathogen-inducible cis-elements or stress-responsive motifs (40–120 bp) to dynamically drive adaptive transcriptomes under stress (Ke et al., 2025). Building on this idea, transcriptional regulators based on dCas9 (dead Cas9) and dCas12 have been engineered by fusing activation or repression domains to permit programmable, reversible, and multiplexed targeting of endogenous target genes in plants. Recent reports have shown that CRISPRa systems specific to synthetic minimal promoters can activate multiple target pathway genes together and can be useful as logic-gated gene circuits in response to developmental or environmental signals (Kang et al., 2024). In combination with modular synthetic promoters and guide RNA libraries, these systems have been effectively implemented to create metabolite-sensing biosensors and feedback-regulated circuits, thus allowing the dynamic regulation of metabolic flux and stress-responsive capabilities. In sugar beet, while dCas9-based transcriptional activation or repression (CRISPRa/i) and synthetic gene circuits are powerful proposed designs, they are yet to be empirically verified in regenerated crops due to tissue culture recalcitrance. Researchers have proposed applying CRISPRa/i tools (such as dCas9-VP64 or dCas9-SRDX) to dynamically tune stress-responsive genes (including the *BvDREB* and *BvWRKY* transcription factor families) or to repress competing vacuolar invertase genes under chilling or drought stress (Keller and Neuhaus, 2025). Furthermore, inserting synthetic enhancers or stress-responsive promoters upstream of the tonoplast sucrose loader *BvTST2;1* is proposed to increase taproot sink strength and maximize industrial sucrose yield without causing vegetative development browning or growth penalties.

### 5.6 CRISPR-based programmable gene circuits in plants

One new direction in plant synthetic biology is programmable gene circuits, which convert specified inputs into predictable biological responses. CRISPR/Cas systems are becoming the technology of choice, serving as the modular wiring system for gene circuits. CRISPR-based circuits, unlike traditional overexpression or knockout strategies, enable layered control with multiple guide RNAs that activate, repress, and implement conditional logic, which can be tuned in response to internal developmental signals or external stresses. An interesting demonstration of this type of programmable control was shown in *Nicotiana benthamiana* and Arabidopsis, where a CRISPR/dCas9 system with synthetic guide RNA cascades was developed to act as Boolean logic gates that integrate multiple environmental signals to control stress-responsive transcription factors; this has shown predictable modulation of both gene expression and downstream physiological responses (Garcia-Perez et al., 2022). In another experiment, a CRISPRi gene circuit was expressed to silence a competing metabolite network branch only upon an abiotic signal (e.g., osmotic stress), thereby constituting a feedback-regulated toggling system that dynamically regulates metabolite flux in planta (Khan and Lister, 2025).

The structural complexity of these plant circuits has been significantly expanded by integrating site-specific recombinases (such as bacteriophage φC31 or Flp) to create stable, memory-based toggles (Lloyd et al., 2022), alongside highly reversible CRISPRi-based logic devices (such as NOR gates) that utilize dCas9 fused to the transcriptional repressor ZAT10 (Khan and Lister, 2025). When translating these advanced genetic computations to sugar beet, programmable circuits offer an essential proposed blueprint to coordinate multi-genic stress adaptations without incurring growth penalties (Gaoua & Arslan, 2026). High-resolution contiguous mapping of the EL10 genome provides a validated sequence platform to design orthogonal CRISPRi and CRISPRa modules that can be wired as synthetic logical AND/OR gates (McGrath et al., 2023).

In sugar beet, these circuits are proposed to integrate soil salinity and cold inputs to dynamically activate endogenous *BvMYB* and *BvDREB* transcription factor networks strictly under extreme environmental conditions, preventing the systemic, constitutive transcriptional browning and yield losses typical of traditional genetic engineering (Gaoua and Arslan, 2026). Such programmable architectures demonstrate the ability of CRISPR technologies to move beyond fixed edits and develop dynamic and adaptive responses to environmental variability in plants, which can establish a basis for precision agricultural characteristics and optimize resource allocation in response to environmental variability.

### 5.7 CRISPR-based biosensors and responsive regulatory networks

CRISPR-based biosensors mark a paradigm shift in plant synthetic biology by enabling the combination of molecular sensing with programmable transcriptional outputs for dynamic, context-dependent modulation of traits. These sensing-response systems use guide RNAs and catalytically inactive Cas proteins to detect endogenous metabolites, signaling molecules, or stress-related signals, thereby activating specific transcriptional programs under synthetic or native promoters. Recent work has demonstrated metabolite-responsive CRISPRa/i circuits that modulate gene expression in real time in response to intracellular levels of flavonoids, terpenoids, or phytohormones, and stress-responsive circuits can alter drought- or salinity-associated transcription factors only under defined environmental conditions, with minimal growth penalties under non-stressful conditions (Hoang et al., 2017). These real-time responsive networks frequently recruit engineered synthetic transcription factors (synTFs), such as hormone-activated Cas9-based repressors (HACRs), which leverage endogenous hormone-responsive degron domains to transiently de-repress target genes in the presence of auxin or abscisic acid (Morey and Khakhar, 2024). In sugar beet, the integration of such biosensors represents a vital proposed pathway to dynamically regulate source-sink relations and sucrose partitioning. To replace traditional, static overexpression constructs, researchers have proposed designing sucrose-sensing synthetic transcription factors that activate the tonoplast sugar transporter *BvTST2;1* strictly as taproot storage tissues expand (Wang and Demirer, 2023; Keller and Neuhaus, 2025). This proposed design would quantitatively coordinate vacuolar sucrose loading on-demand, bypassing the developmental bottlenecks of tissue culture and minimizing cold-induced sweetening in taproots during post-harvest storage (Gaoua and Arslan, 2026). These biosensors can be combined with synthetic promoter libraries and logic-gated circuits to produce multiplexed, programmable outputs, such as conditional activation of metabolic pathways or feedback-controlled stress resilience. Notably, these CRISPR-derived sensing networks can be delivered using tissue culture-free vectors, such as RNP complexes or viral vectors, enabling rapid in planta deployment without transgenic integration and, therefore, supporting precision agriculture strategies that dynamically regulate resource allocation, metabolite production, and environmental adaptability without causing significant environmental impact.

### 5.8 Ethical and biosafety perspective in CRISPR-based plant synthetic biology

As CRISPR-enabled synthetic biology advances toward programmable circuits, synthetic promoters, and metabolite-responsive networks, it is imperative to proactively address ethical implications, environmental concerns, and biosafety in the use of synthetic biology for food production. Transgene-free technologies such as ribonucleoprotein (RNP) delivery and tissue culture-free GE reduce regulatory complexity and minimize unintended genomic integration, but synthetic rewiring of metabolic and signaling networks may introduce metabolic burdens, growth effects, or ecological interactions, posing potential risks to fitness, pollinator interactions, or horizontal gene transfer (Bhattacharjee et al., 2023). Globally, the regulatory classification of such edited crops depends heavily on whether they are categorized as SDN1 (template-free modifications), SDN2 (precise base edits), or SDN3 (large-fragment transgene insertions), with countries like the United States, Japan, the Philippines, and the United Kingdom adopting lighter-touch, product-based exemptions for transgene-free edits, while the European Union maintains a strict, process-based GMO classification (Ahmad, 2023; Tachikawa and Matsuo, 2024; Lokya et al., 2025). This regulatory fragmentation poses significant barriers to international trade and commercialization, requiring researchers to prioritize precise, DNA-free editing platforms to minimize unintended off-target edits or residual vector backbones in food crops (Wolt et al., 2016; Ahmad, 2023). Risk assessment is further complicated by programmable circuits, where conditional and feedback-controlled systems may behave unpredictably in field conditions, requiring careful case-by-case analysis of both planned and unintended impacts. Community acceptance and management are paramount, especially when plants are designed to produce elevated levels of metabolites or to be resistant to environmental stress; thus, effective communication, active stakeholder inclusion, and harmonized regulatory policies are essential. Together, responsible innovation in plant synthetic biology involves integrating stringent biosafety evaluation, ecological controls, and clear regulatory oversight to enable CRISPR-enabled metabolic and regulatory rewiring to be used sustainably, safely, and ethically in agriculture (Bohua et al., 2023).

In *B. vulgaris*, navigating this ethical and biosafety landscape highlights a stark divide between demonstrated transgene-free edits and proposed synthetic biology circuits. Simple, template-free SDN1 modifications, such as knocking out the host susceptibility factor *Bv-eIF(iso)4E* to establish recessive BChVresistance, represent actively demonstrated, low-risk edits that qualify for regulatory exemption under the SECURE rule of USDA (Hoffman, 2021; Rollwage et al., 2024). In contrast, proposed future designs, such as multiplexed logic circuits, synthetic promoters, or the horizontal gene transfer of cross-kingdom microbial genes to establish C4-like photosynthetic efficiency, fall under transgenic or SDN3 regulatory frameworks, necessitating extensive multi-season field validation, metabolic profiling, and transparent biosafety evaluations to secure public trust and market feasibility (Ahmad, 2023; Wang and Demirer, 2023).

## 6 The future of new technologies: beyond CRISPR

Despite the ease with which the CRISPR/Cas9 system has enabled the engineering of plant genomes on a large scale, the system faces major limitations. The size of approximately 1368 amino acids (~160 kDa), PAM dependency, and cytotoxic DSBs are among the major limitations of the most widely used *Streptococcus pyogenes* Cas9 effector. Owing to these factors, it is less useful in general, especially for viral delivery and precise GE (Son and Park, 2022; Wang et al., 2024). Therefore, there have always been efforts to improve its utility, and this can be regarded as a post-Cas9 age. This emerging group of technologies is characterized by the derivation of ancestral transposon-encoded nucleases (the OMEGA superfamily), eukaryotic RNA-guided endonucleases, and programmable RNA editors. Their key features are miniaturization, unlimited targetability, and scar-free accuracy, which together expand the range of tools available for improving plants, compared to traditional mutagenesis (Zess and Begemann, 2021; Ben Amar, 2024). In sugar beet, these constraints are particularly acute. The bulky size of SpCas9 and its strict NGG PAM requirement severely restrict targeting flexibility, particularly within the AT-rich regulatory regions and promoters of key stress-responsive genes, while also preventing the packaging of the complete editing machinery into transient, space-constrained delivery vehicles such as Tobacco Rattle Virus (TRV) vectors or compact nanocarriers (Miroshnichenko et al., 2026). Consequently, transitioning to hypercompact, altamente specific, and PAM-relaxed post-Cas9 editing tools is actively proposed as a key strategy to enable tissue-culture-independent, transgene-free breeding directly in elite commercial sugar beet lines (Gaoua and Arslan, 2026).

### 6.1 Hypercompact nucleases

The main limitation to successful in-planta delivery is the size of the cargo of viral vectors, which often renders the encapsulation of standard Cas9 nucleases impossible. To overcome this constraint, transposon-encoded proteins (e.g. Prokaryotic TnpB and eukaryotic Fanzors), the evolutionary ancestors of the Class II CRISPR-Cas12 family, have been employed successfully. Transposon-associated protein B (TnpB) has become a paradigm-shifting technology; these nucleases are programmable to cleave DNA at a specific site, typically at sequences flanking a Transposon-Associated Motif (TAM), and generally are 350 to 500 amino acids in size, approximately 1/3rd the size of SpCas9 (Karvelis et al., 2021; Xu et al., 2023). Recent developments have increased the rate of adaptation of this prokaryotic potential to powerful plant-editing platforms. Deinococcus geothermalis TnpB variant efficiently developed edited rice lines. The similar success has been documented in monocots and dicots (Zhang et al., 2024; Karmakar et al., 2025). The small size of TnpB has also made it possible to implement new delivery approaches; Weiss et al. (2025) have managed to insert the ISYmu1 TnpB variant into a Tobacco Rattle Virus (TRV) virus (Weiss et al., 2025). The small size of the enzyme makes it easy to encapsulate it into viral capsids without limiting systemic transport, thus making heritable editing in Arabidopsis possible, which was previously limited by cargo size (Shen et al., 2024). Similarly, AsCas12f from the CRISPR/Cas12f system has also been delivered into tomato and *N. benthamiana* through the same viral vector (Hu et al., 2025). In order to further enhance the efficiency of editing, Marquart et al. (2024) used deep-learning algorithms to engineer TnpBmax, an improved version featuring an increased TAM recognition, which increased the activity of the protein by 4.4-fold compared to wild-type enzymes (Marquart et al., 2024). Furthermore, research on other native TnpB variants (such as ISDra2 and ISYmu1) has demonstrated highly efficient targeted mutagenesis in crops when expressed under plant-specific promoters, achieving editing efficiencies up to 33.58% in rice and heritable mutations in Arabidopsis and various medicinal species (Li et al., 2024; Karmakar et al., 2025). In sugar beet, deploying prokaryotic TnpB represents a premier proposed strategy for transient, viral-mediated *in planta* delivery. By packaging TnpB and its right-end element (reRNA) into phloem-limited viral vectors, such as *Beet necrotic yellow vein virus* (BNYVV) or deconstructed geminiviruses, breeders propose to deliver editing reagents systemically to the shoot apical meristem of bolting crowns, enabling heritable targeted mutagenesis of agronomic loci (e.g., the bolting-regulator *BvFT2*) without T-DNA integration or a sterile tissue culture phase (Jiang et al., 2019; Kocsisova and Coneva, 2023).

At the same time, the identification of Fanzors (Fz), eukaryotic TnpB homologs in algae, fungi, and molluscs (Saito et al., 2023) does not support the idea that only prokaryotes can cleave with the help of RNA. Fanzors are eukaryotic origin, chromatin-sensitive, RNA-guided nucleases having inherent nuclear localisation signals (Jiang et al., 2025). Another notable benefit of Fanzors compared to most prokaryotic systems is that there is no collateral cleavage activity and, therefore, minimal nonspecific degradation of DNA and resultant cytotoxicity. Optimized versions, such as SpuFz1-v2, have a maximum indel activity in human cells, suggesting that Fanzors can be employed as a native and low-toxicity alternative to transplantation of bacterial immune systems into plant nuclei (Saito et al., 2023). Although Fanzor editing has been successfully demonstrated in mammalian cells (Ji et al), its adaptation to plant synthetic biology remains in the earliest proposed stage, with no published reports of Fz-mediated genome editing in crop species (Yaşar et al., 2026). For highly recalcitrant crops like sugar beet, eukaryotic Fanzors are proposed as a highly biocompatible, low-toxicity alternative to bacterial nucleases, as their lack of collateral cleavage activity is expected to prevent the severe somatic cytotoxicity and cellular necrosis frequently observed during the transfection of classical Cas9 or Cas12a reagents in sugar beet callus tissues (Gaoua and Arslan, 2026).

### 6.2 Transcriptome engineering

Despite the permanence of DNA edits, they are highly prone to off- target mutagenesis and face regulatory hurdles, especially when only a transient phenotypic change is needed, creating a push toward transcriptome engineering. Transcriptome engineering offers a reversible approach to altering expression without changing genomic sequences, thereby fixing a point mutation or controlling protein function and physiology. Generating transgenic organisms is not seen as desirable by many critics, and therefore RNA editors are the best alternatives for rectifying deleterious mutations involved in key traits (Zhang et al., 2024). In plant biotechnology, Cas13-based platforms (such as the REPAIR or RESCUE systems) have already been engineered by fusing catalytically inactive dCas13 to modified deaminases, demonstrating precise, transient A-to-I and C-to-U transcript editing in eukaryotic systems (Cox et al., 2017; Abudayyeh et al., 2019). For a biennial root crop like sugar beet, where permanent genomic changes trigger strict European process-based GMO regulations, transient RNA-level edits offer an attractive proposed alternative to regulate key agronomic traits, such as suppressing vacuolar invertases during post-harvest storage to prevent sucrose loss, without altering the hereditary blueprint of the plant (Keller and Neuhaus, 2025).

The LEAPER system is the most representative of such a paradigm shift. LEAPER requires the introduction of a small, synthetic RNA molecule, unlike CRISPR systems, which require large exogenous Cas nucleases. The ADAR -recruiting RNA (arRNA) hybridizes with a target mRNA, creating a double- stranded RNA (dsRNA) intermediate that mimics the natural substrate of Adenosine Deaminase Acting on RNA (ADAR) enzymes, ubiquitous in eukaryotes. When recruited, endogenous ADAR catalyzes the deamination of adenosine (A) to inosine (I) at the desired locus, thus causing a precise A- to- G substitution in the translated protein without affecting the genomic DNA, since inosine is translated as guanine (G) (Qu et al., 2019b; Aquino-Jarquin, 2020a).

Although the concept had strong appeal, the initial LEAPER designs that used linear antisense RNAs had poor degradation rates in the cellular environment and low editing efficiencies. To overcome this hurdle, circular arRNAs (circ- arRNAs) were introduced in place of linear RNAs in LEAPER 2.2.0. Covalent circularization also increases resistance to exonuclease- mediated degradation, which significantly increases the half- life of the guide RNA *in vivo* (Ruden, 2025). CircRNAs exhibit an average 3.1-fold enhancement in editing activity relative to their linear counterparts. Circular LEAPER systems are biologically contained and do not require exogenous protein production, thus decoupling genome engineering from the expression of foreign proteins and avoiding numerous biosafety and regulatory challenges associated with conventional GMOs (Yi et al., 2022). However, the practical application of LEAPER in plants is currently constrained by a major physiological limitation. Although ADAR-like enzymes are ubiquitous in metazoans, there is currently no evidence demonstrating the presence or active functioning of endogenous ADARs in plant systems (Merkle et al., 2019; Aquino-Jarquin, 2020b). Consequently, to implement LEAPER in sugar beet, researchers have proposed co-delivering arRNAs alongside transgenic or transiently expressed human ADAR1 or ADAR2 catalytic domains, or utilizing *de novo* designed plant-compatible RNA deaminases. If successfully established, this proposed strategy would enable the transient correction of susceptibility transcripts, such as those involved in *Cercospora beticola* or BNYVV susceptibility, providing robust crop protection in the field while completely bypassing the environmental and biosafety restrictions imposed on transgenic crops (Gaoua and Arslan, 2026).

### 6.3 Unlimited targeting systems

Despite solutions to delivery and reversibility problems through miniaturization and RNA editing, a fundamental biochemical constraint remains, the protospacer adjacent motif (PAM). The specificity of Cas nucleases for PAM sequences (e.g., NGG with SpCas9) renders large sections of the plant genome, often referred to as genomic dark matter, uneditable. This limitation is particularly significant in AT-rich regions. Therefore, there has recently been a shift from engineering Cas variants to discovering novel RNA-guided scaffolds. To alleviate this PAM bottleneck, researchers have engineered SpCas9 variants with broadened targeting ranges, such as SpG (recognizing NGN PAMs) and the near-PAMless SpRY (recognizing NRN and NYN PAMs), which have been demonstrated to target almost 100% of genes in staple crops (Walton et al., 2020). Concurrently, Type V Cas12a (Cpf1) orthologs (such as LbCas12a and AsCas12a) recognize T-rich PAM sequences (5’-TTTV-3’) and catalyze staggered cuts, expanding target accessibility in AT-rich regions where SpCas9 is ineffective (Malzahn et al., 2019; Yao et al., 2024). In sugar beet, whose 758-Mb genome has been contiguously mapped in the EL10 germplasm, AT-rich motifs are highly prevalent in the promoters and enhancers of critical disease-resistance and sucrose-metabolizing genes (McGrath et al., 2023; Gong et al., 2025). Employing temperature-tolerant LbCas12a or engineered SpRY-based base editors is an actively demonstrated strategy in sugar beet protoplast screens, allowing breeders to edit previously inaccessible “genomic dark matter” sites with high precision and minimal off-target risks (Rollwage et al., 2024).

Among the achievements in this field, the characterization of TIGR-Tas (Tandem Interspaced Guide RNA-Targeting Systems) stands out. As shown in **Figure 2**, the TIGR-Tas is a specialized form of RNA-guided enzymes that does not rely on the CRISPR-Cas mechanism (Ruden, 2025). TIGR-Tas uses a dual-spacer tigRNA to interrogate both strands of the DNA helix concurrently, unlike Cas effectors, which interrogate only one strand of the DNA helix using a single guide RNA. Theoretically, this bipartite recognition system allows the effector protein to interact with any genomic locus in plants without the need for a conserved PAM sequence. Furthermore, TIGR-Tas produces different 8-nucleotide 3’ overhangs, a sticky-end format that is not similar to the blunt ends of Cas9 or the staggered ends of Cas12. Therefore, undividing cells, where Homology-Directed Repair (HDR) is stopped, can also be edited (Wang et al., 2024). Although TIGR-Tas represents a highly promising RNA-guided DNA-targeting system originally identified in prokaryotes and their viruses (Faure et al., 2025), its translation to plant systems remains a strictly proposed, future blueprint. If successfully adapted to the genus *Beta*, the dual-spacer tigRNA architecture would allow researchers to bypass PAM constraints entirely and coordinate precise, sticky-ended edits directly in the non-dividing, highly specialized cells of the mature taproot (Faure et al., 2025). This capability would enable the direct, scarless knock-in of beneficial alleles, such as drought-responsive enhancers or cold-stabilizing desaturases, without requiring active cell division or the delivery of complex homologous donor templates.

**Figure 2:**
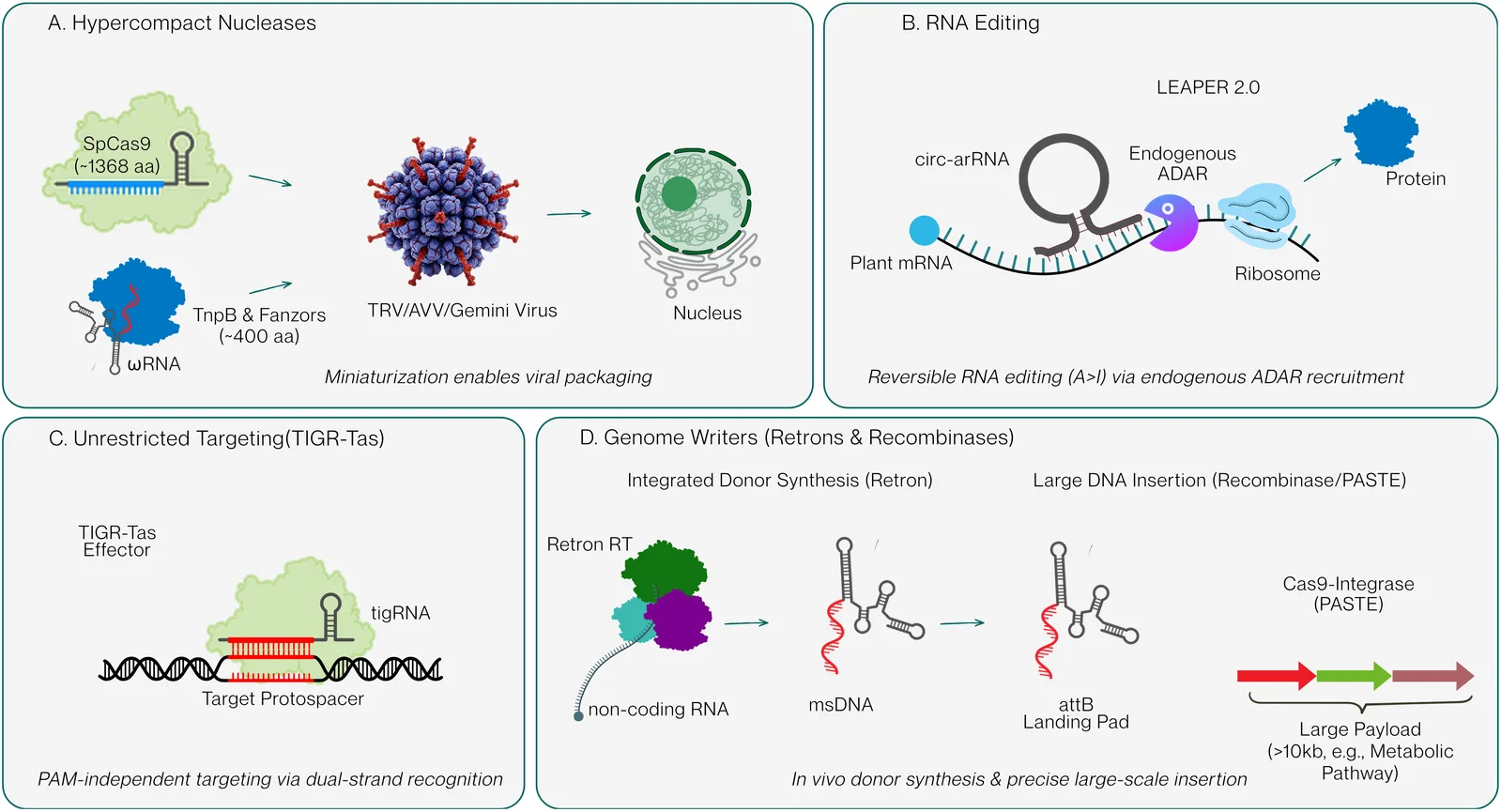
The Post-Cas9 toolbox of plant genome engineering. **(A)** Miniaturisation: Relative analysis of large SpCas9 ~1368 aa and hypercompact TnpB/Fanzor nucleases ~400 aa. TnpB is effectively encapsulated in a viral capsid (such as TRV) in which Cas9 cannot be packed. **(B)** Transcriptome engineering: LEAPER 2.0 mechanism. The target mRNA is recruited by a circular ADAR-recruiting RNA (circ -arRNA) to a stable duplex with the endogenous ADAR, which catalyzes a specific Adenosine-Inosine (A><I) base change. **(C)** Unrestricted targeting: TIGR-Tas complex is using a dual-spacer RNA in the process of identifying both strands of DNA simultaneously, thus eliminating the requirement of a specific PAM sequence. **(D)** Genome writing: A Retron element expresses a single stranded DNA (msDNA) donor *in vivo* which is a template for Homology-Directed Repair (HDR) or recombinase mediated insertion of large clusters of genes.

### 6.4 Genome writers

The final goal of plant metabolic engineering extends beyond gene disruption to include the precise insertion of intricate biochemical pathways (gene stacking) and synthetic circuits. Although Cas9 functions as molecular scissors, it is a poor genome writer, relying on the host cell’s error-prone repair systems to insert foreign DNA, and its efficiency decreases with cargo size. To bridge this complexity divide, innovative technologies are emerging as real genome writers, assembling and recombining donor templates within the editing machinery (**Figure 3**). While traditional homology-directed repair (HDR) facilitates exact sequence replacement, its historically low efficiency in plants has driven the development of hybrid approaches like tandem repeat-HDR (TR-HDR), which leverages initial non-homologous end joining to create an internal repair template (Muzaffar et al., 2026). In sugar beet, precise genome writing via traditional HDR is heavily bottlenecked by exceptionally low homologous recombination frequencies in somatic cells. While hybrid NHEJ/HDR strategies like TR-HDR are proposed to bypass this limitation by using *in vivo* linearized repeat templates to increase local donor availability, their empirical application in the genus *Beta* remains completely unverified (Schmidt et al., 2019; Gaoua and Arslan, 2026; Miroshnichenko et al., 2026).

**Figure 3:**
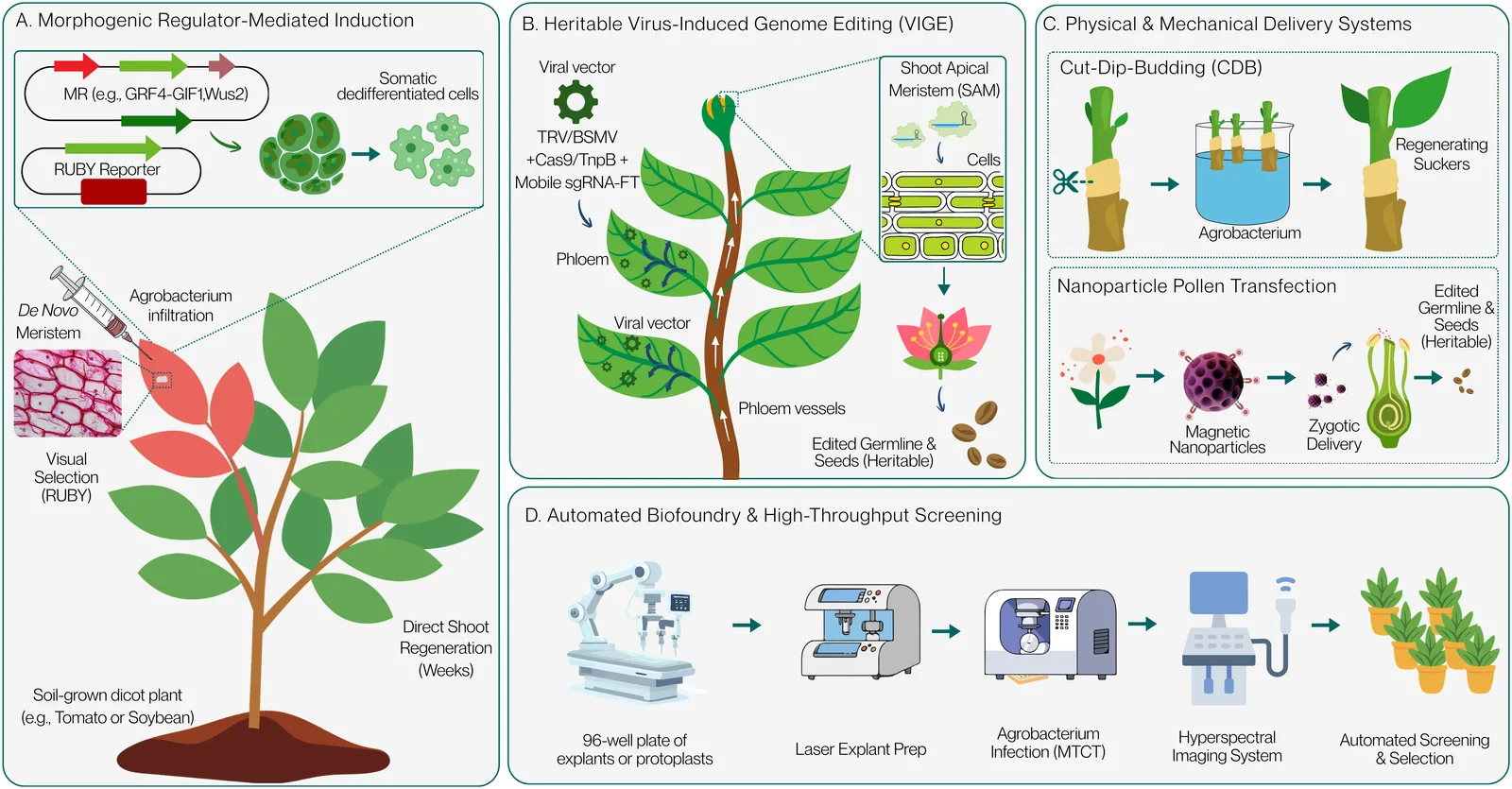
Overcoming the tissue culture bottleneck strategies. **(A)** Transcriptional Reprogramming: Co-delivery of CRISPR reagents and Morphogenic Regulators (e.g., GRF4-GIF1 chimeras) via Agrobacterium. Gene-edited shoots (purple pigmentation) that grow directly from the soil-grown plant node can be visually identified by including the RUBY reporter. **(B)** Heritable Virus-Based Genome Editing (VIGE): A viral vector (e.g., TRV or PVX) engineered with a mobile single-guide RNA (sgRNA conjugated to FT mRNA). The mobile signal transports the editing complex into the phloem and to the Shoot Apical Meristem (SAM), leading to Cas9 activity in the germline and the generation of edited seeds. **(C)** Physical Delivery (Cut-Dip-Budding): The apical shoot is removed to induce root suckering. The injured surface is inoculated with Agrobacterium, and the root system’s natural regenerative ability generates transformed buds without exogenous hormones. **(D)** Industrial Automation: A Biofoundry workflow uses robotic liquid handlers (e.g., Opentrons) to normalize protoplast isolation or Agrobacterium infection in multi-well plates, enabling high-throughput screening of Minimal Tissue Culture (MTCT) events.

A major challenge in accurate HDR editing is that exogenous DNA donor templates are often degraded by cellular nucleases before use. Retrons offer a biological solution: bacterial retroelements comprising a reverse transcriptase and a non-coding RNA that serves as a copy of the donor template in the target cell. Jiang et al. (2022) transferred the prokaryotic Eco1 retron system to *N. benthamiana*, yielding high-titer, multicopy single-stranded DNA (msDNA) in the plant nucleus (Jiang et al., 2022). The msDNA generated *in vivo* is a strong, stable HDR donor and more efficient than exogenous oligonucleotides for gene targeting. Later, Crawford et al. (2025) applied machine-learning algorithms to elucidate the design principles of retrotransposon non-coding RNAs, enabling the automated generation of high-activity retron libraries (Crawford et al., 2025). This integration of synthetic biology with artificial intelligence enables large-scale screening of variant libraries, effectively transforming plant cells into autonomous editing factories (Kaur and Pati, 2024). For highly recalcitrant crops like sugar beet, establishing *in vivo* retron-mediated msDNA synthesis represents a promising proposed strategy to protect templates from endogenous nucleases. However, translating these multi-component prokaryotic systems into the genus *Beta* remains strictly in the proposed phase, as it is constrained by the need for meticulous codon optimization to prevent translation initiation shifts and cellular cytotoxicity in highly heterozygous genetic backgrounds (Song et al., 2025).

In addition to donor generation, Site-Specific Recombinases (SSRIs) enable the insertion of large DNA cargoes (>1015 kb), such as multi-gene clusters for metabolites, without causing lethal, unrepaired double-strand breaks. Massive serine integrases, such as Bxb1, are increasingly recruited to Cas effectors (e.g., the PASTE system (Programmable Addition via Site-specific Targeting Elements)), where a genome-specific landing pad (attB site) is inserted to achieve targeted integration. Building on this, modern platforms such as PrimeRoot unite prime editing with site-specific recombinases to achieve double-strand break-free integration of large payloads up to 11.1 kb. This facilitates the targeted placement of transgenes into genomic ‘SafeHarbor’ regions, significantly streamlining downstream trait stacking. Additionally, transposable element platforms like transposase-assisted target-site integration (TATSI), which utilizes the rice *mPing* element, have expanded precise insertion capabilities up to 8.6 kb. Furthermore, exploiting naturally occurring mobile elements, such as rolling-circle helitron transposable elements, offers an untapped route for gene duplication with minimal genomic footprints (Gilbertson et al., 2025). Orthogonal LoxPsym sites have also increased the usefulness of recombinases. To enable multiplexed gene stacking, i.e., transgene insertion at different genomic loci without cross-reactivity, Cautereels et al. (2024) established a toolkit of such variants (Cautereels et al., 2024). More advanced synthetic biology programs build on this technology, enabling the assembly of complex genetic circuits, including those needed to enable C4 photosynthesis or nitrogen fixation, within the plant genome itself (Lloyd et al., 2025). In sugar beet, implementing these advanced genome writing platforms represents an essential proposed roadmap to bypass traditional tissue culture, outcrossing self-incompatibility, and the 2-year biennial cycle. While the PrimeRoot platform has successfully inserted up to 11.1 kb into safe harbors in monocots (Sun et al., 2024), and the TATSI system has demonstrated up to an 18.2% targeted integration rate for gene-scale cassettes in soybean (Liu et al., 2024), no such precise macromolecular integrations have been demonstrated in sugar beet. Utilizing high-resolution contiguous EL10 genomic mapping to predict safe harbors, researchers have proposed employing PrimeRoot or TATSI systems to stack multi-genic agronomic traits directly into elite heterozygous clonal germplasms via non-integrating viral vectors or *in planta* shoot meristem injections (McGrath et al., 2023; Keller and Neuhaus, 2025). This proposed design would achieve stable, highly coordinated trait expression while completely avoiding chromosomal insertions of vector backbones or marker genes, fulfilling both public interest and global biosafety regulations. Looking ahead, translating other high-efficiency insertion systems established in mammalian or bacterial models, including CAST and SAGE, presents a highly promising frontier for expanding the plant synthetic biology toolkit (Muzaffar et al., 2026).

### 6.5 Targeted large-fragment DNA knock-in

The field of plant genome engineering is changing rapidly, from simple gene disruption to the precise programming of complex genomic architectures (Zhang et al., 2025; Yaşar et al., 2026). Although the initial applications of CRISPR were for small insertions or deletions, recent advances have created a powerful toolbox for the targeted knock-in (KI) of gene-sized and larger DNA fragments (Sun et al., 2025; Zhao et al., 2025). These advances are crucial for engineering multi-genic traits, reconstructing whole metabolic pathways, and for the assembly of plant artificial chromosomes.

#### 6.5.1 Recombinase-based large-scale integration

The recent development of Prime Editing (PE) and Site-Specific Recombinases (SSRs) is one of the most important advances, as it overcomes the limitations of both systems. PE is very precise but can only be used for small edits, whereas SSRs can be used to integrate large sequences but need to be pre-installed with recognition sites, and when combined, they can integrate large sequences without double-strand break (DSB) (Zhang et al., 2025; Yaşar et al., 2026). One of the first examples is the PrimeRoot system. This has been further optimized by Programmable Chromosome Engineering (PCE) that uses AI-driven engineered recombinases for kilobase to megabase scale manipulations. Notably, the RePCE variant can be used for “scarless” editing, where residual recombination sites are restored to the original genomic sequence after integration using a specially designed editor (Sun et al., 2025). Moreover, the Kp03 large serine recombinase has been shown to be highly efficient for the integration of donors up to 27.3 kb in rice and Arabidopsis protoplasts (Yan et al., 2025).

In sugar beet, recombinase-based writing remains strictly proposed: genetic engineering here has so far relied only on Agrobacterium-mediated random T-DNA integration of single genes, such as *1-sst* (fructan synthesis) or *SacB* (drought tolerance), and CRISPR editing is limited to standard NHEJ knockouts (*BvMgCh*, *BvPDS*) (Pilon-Smits et al., 1999; Weyens et al., 2004; Khan et al., 2025). Site-specific integration via PrimeRoot or Kp03 faces the same bottleneck as HDR in which large donor plasmids transfect into protoplasts, but regenerating whole plants from them is unreliable across commercial germplasm. Two translational targets have been proposed; stacking a fructan-biosynthesis pathway (*1-sst, 6g-fft*) into a genomic safe harbor identified from the EL10 reference genome (McGrath et al., 2023; Yan et al., 2025), and knocking in inducible morphogenic regulators (*BvBBM*, *BvWUS*) to build a chemically triggered regeneration switch (Youngstrom et al., 2025).

#### 6.5.2 RNA-guided transposition systems

Another frontier is the use of CRISPR-associated transposons (CASTs) and artificially engineered transposase systems (Liu et al., 2024; Zhao et al., 2025). These technologies combine the RNA-guided targeting ability of CRISPR with the high-capacity integration machinery of transposable elements (Ali et al., 2025). One of the most significant advances is the combination of the rice-derived Pong DNA transposon with Cas9 or Cas12a nucleases, known as TATSI system that has been shown to achieve targeted integration efficiencies of up to 18.2% in soybean. In addition to TATSI, hypercompact nucleases such as TnpB have been discovered, providing new possibilities for viral delivery of large-fragment editing tools because of their small size (Ali et al., 2025). RNA-guided transposition in sugar beet remains an unverified blueprint: the genome is rich in endogenous LTR retrotransposons and DNA transposons, but harnessing them for programmable insertion has not been demonstrated in the genus Beta (Zakrzewski et al., 2017; Gürel et al., 2025). A TATSI-style system is proposed to replace random Agrobacterium integration by transiently delivering transposase-Cas complexes to somatic tissue such as blade-petiole transition zones, avoiding permanent nuclease persistence (Zhang et al., 2024).

#### 6.5.3 The expansion of the limits of prime editing

Although the original PE could only be used to insert up to 50 base pairs, methodological advances have significantly expanded the range of possible insertions (Anzalone et al., 2019). Dual prime editing (DualPE) is a method that uses two engineered pegRNAs to create complementary 3’ DNA flaps for seamless large-scale modifications such as sequence replacement up to 258 kb and deletion up to 2 Mb in wheat, tobacco, and tomato (Yaşar et al., 2025; Zhao et al., 2025). Moreover, the natural mechanism of retrotransposons has been optimized to insert fragments up to 710 bp in rice, called Template-Jumping Prime Editing (TJ-PE). This method only needs one TJ-pegRNA and one nicking sgRNA, which is simpler than the dual-pegRNA method for medium-sized insertions (Muzaffar et al., 2026). Base editing, DualPE and TJ-PE remain proposed, not demonstrated, in the genus *Beta*. Candidate applications include rewriting susceptibility-gene exons for rhizomania and nematode resistance, replacing the *BvSUT4* promoter to curb premature bolting and post-harvest sucrose loss, and editing *Rf* loci for CMS breeding (Pfeiffer et al., 2014; Misra et al., 2024; Zhong et al., 2025).

#### 6.5.4 Enhanced DSB-dependent strategies

Precision and efficiency have also been significantly enhanced for researchers who still want to use DSB-repair pathways with cleavable donor designs and nuclease engineering (Schreiber et al., 2024; Gu et al., 2025). Successful knock-in of fragments larger than 10 kb has been reported in moss (*Physcomitrium patens*) with efficiencies up to 65.6% using *In Vivo* Cleavable (IVC) donor plasmids that only release a linear template when Cas9 is present. Additionally, fusing Cas9 to 5’→3’ exonucleases (e.g., from herpes virus or T7 bacteriophage) has been shown to increase homology-directed repair (HDR) efficiency by up to 38-fold in tobacco and 10-fold in Arabidopsis by creating long 3’ single-stranded DNA overhangs that facilitate strand invasion (Schreiber et al., 2024). A sequential transformation strategy in Arabidopsis also employs the use of Cas9 expression in the egg cell to capture transiently available T-DNA at targeted DSBs, with multi-kilobase integration efficiencies of ~30% (Xu et al., 2025).

#### 6.5.5 Bridge RNA and scarless engineering

In the future, the discovery of Bridge RNA (IS110 family) could be a paradigm-shifting tool in plant biotechnology (Durrant et al., 2024). These “cut-and-paste” elements use a structured non-coding RNA with two programmable loops to recognize the target site and the donor sequence independently, allowing for programmable recombination without inducing DSBs. Primary literature across the related IS110 and IS1111 insertion families terms these transcripts seeker RNAs (seekRNAs), demonstrating that they utilize short internal segments matching both the top and bottom strands of the target DNA to guide orientation-specific transposition. The structural order of these guide matches is reversed between the two families, and redirection requires simultaneously engineering both the seekRNA loops and the target-derived sequences flanking the cargo (Siddiquee et al., 2024). Currently validated in bacteria and human cells for mobilizing large DNA fragments, adapting Bridge RNA to plant systems would allow for complex, scarless genome rearrangements and large-scale path-engineering with unprecedented control. Bridge RNA has not been tested in any plant and remains a proposed blueprint for sugar beet specifically, where low transformation rates, genotype dependency, and random T-DNA integration currently drive costly, process-based GMO regulation. A DNA-free recombinase-Bridge RNA complex delivered transiently to shoot apical meristems is proposed as a route to scarless, regulation-light metabolic edits in elite heterozygous lines (Su et al., 2026). While each targeted integration framework offers distinct capabilities for plant genome engineering, selecting the optimal strategy requires balancing payload size, knock-in efficiency, junction precision, and delivery constraints. A comprehensive comparative synthesis of these DSB-dependent and DSB-free targeted DNA insertion systems is summarized in **Table 3**.

**Table 3:** Comparative performance, repair mechanisms, and delivery constraints of targeted large-fragment DNA knock-in technologies in plants.

Technology	Homology-directed repair (HDR)	NHEJ-based approaches	Prime editing + recombinase (PrimeRoot, RePCE)	Transposase systems (CASTs, TATSI)	Extended prime editing (DualPE, TJ-PE)
Mechanism & DSB Requirement	Cas-induced DSBs repaired via homologous recombination (Chen et al., 2024; Singh et al., 2025).	DSB-dependent donor ligation (Chen et al., 2024).	Non-DSB nickase installs landing sites for integrase capture (Rozov and Deineko, 2025; Sun et al., 2025).	RNA-guided transposition (TATSI relies on DSB) (Tou et al., 2023; Van Vu et al., 2025).	DSB-free nickase using paired/jumping guides (Anzalone et al., 2019; Zheng et al., 2023).
Cargo / Insertion Size Capacity	Up to 11.1 kb (Van Vu et al., 2025; Yaşar et al., 2026).	Best for small-to-moderate inserts(1 bp to 1 kb) (Van Vu et al., 2025).	Up to 11.1 kb (PrimeRoot) or 18.8 kb (RePCE) (Sun et al., 2025; Yaşar et al., 2026).	Up to 8.994 kb in plants (Chen et al., 2024).	~1 kb (paired) to 403–710 bp (TJ-PE) (Zheng et al., 2023; Chen et al., 2024).
Knock-in Efficiency	Low and variable; ~0.24%–2.4% in Arabidopsis (Liu et al., 2024).	Highest practical efficiency; up to 71.75% in cereals/dicots (Van Vu et al., 2025).	6.3% precise integration in rice (PrimeRoot) (Sun et al., 2024; Rozov and Deineko, 2025).	27% (small cargo) to 8.3% (~9 kb) in Arabidopsis (Liu et al., 2024; Yaşar et al., 2026).	2.1%–11.1% precise in rice; higher for partial inserts (Li et al., 2025).
Precision & Junction Accuracy	Highest precision; typically scarless junctions.	Lower precision; frequent small indels or scars.	Highly precise, though recombination scars remain unless engineered away (Van Vu et al., 2025).	Intermediate precision; plant junctions contain scars (Tou et al., 2023; Van Vu et al., 2025).	Lower for larger inserts due to truncations and indels (Li et al., 2025).
Unintended Modifications & Risks	DSB toxicity and low recovery (Anzalone et al., 2019; Rozov and Deineko, 2025).	Scar sequences and imprecise junctions (Anzalone et al., 2019).	Multicomponent system complexity (Sun et al., 2024).	Off-target insertions, donor inversion, and spontaneous DSB capture (Strecker et al., 2019; Tou et al., 2023).	Incomplete template copying and heterogeneous outcomes (Chen et al., 2024; Li et al., 2025).
Delivery & Tissue Culture Constraints	Complex donor design with homology arms; highly tissue-culture dependent (Muzaffar et al., 2026).	Simpler donor design, but still relies on transformation workflows (Van Vu et al., 2025).	Bottlenecked by large multigene constructs; constrained by transformation (Negi et al., 2026).	TATSI allows one-step delivery but remains tissue-culture dependent (Lampe et al., 2024; Van Vu et al., 2025).	Delivery limited by large PE expression cassettes (Zhao et al., 2025).

## 7 Genome editing bottlenecks: tissue culture barrier

Despite the unprecedented precision of next-generation systems across multiple categories, their deployment in crop improvement remains anchored in a decades-old delivery paradigm: *in vitro* tissue culture. For most agriculturally important species, especially elite cultivars of cereals, legumes, and woody perennials, the transformation process is the single most important rate-limiting step. For instance, the tissue culture barrier is particularly problematic for sugar beet, which is notoriously recalcitrant to *in vitro* regeneration and transformation. Conventional *Agrobacterium*-mediated transformation in sugar beet often yields incredibly low efficiencies and suffers from severe genotype dependency (Sun et al., 2025; Miroshnichenko et al., 2026). This dependence on the dedifferentiation of somatic cells into callus and their regeneration into entire plants is stochastic, laborious, and highly genotype-specific (Liu et al., 2024; Sun et al., 2024). Moreover, prolonged culture often induces somaclonal variation, leading to unintended mutations in the genome and epigenome that can mask or compromise the desired edited phenotype. As a result, the discipline is currently experiencing a paradigm shift, with optimization of culture media moving toward the total elimination of culture media. This shift to Tissue Culture-Independent (TCI) or *In Planta* approaches has enabled the use of Petri plates or artificial media that rely on the plant’s own regenerative mechanisms. This part provides a critical analysis of the four main frontiers that are driving this revolution: transcriptional reprogramming, viral persistence, physical delivery, and automated industrialization.

### 7.1 Morphogenic regulators and *de novo* meristem induction

The stochastic and genotype-specific nature of the delivery and regeneration process is currently limiting the universal applicability of plant genome engineering, not by the accuracy of the editing machinery but by variation in regeneration processes across families and down to the species level. The canonical transformation workflow, which uses hormones to turn somatic cells into callus, is labor-intensive and likely to cause somaclonal variation. This makes it hard to use precision breeding on elite cultivars of cereals, legumes, and woody perennials (Scheben and Edwards, 2018; Bekalu et al., 2023). To overcome this bottleneck, the field has shifted to so-called TCI paradigms. This method uses genetically encoded Morphogenic Regulators (MRs) to directly reprogram somatic cells in the plant into shoot apical meristems (SAMs), taking advantage of the plant’s natural ability. This shortens the time it takes to make new plants from months to weeks (Maher et al., 2020; Zhang, 2020).

Early studies had already determined that the ectopic expression of certain transcription factors, including those of the homeodomain protein WUSCHEL (WUS), the KNOTTED1-like homeobox protein SHOOT MERISTEMLESS (STM), and the cytokinin-biosynthetic gene Isopentenyl Transferase (IPT), can induce the reversion of differentiated somatic cells into a pluripotent stem-cell state (Maher et al., 2020; Indibi et al., 2025). This idea was implemented in a method called Fast-TrACC (Fast-Treated Agrobacterium Co-Culture), in which Agrobacterium tumefaciens is used to co-deliver these regulators together with the reagents for the CRISPR/Cas system into the axillary nodes of soil-grown plants. *De novo* meristems resulting from the transient expression of WUS2 and STM grow into genetically distinct plants. This approach has also been applied to the model crop *N. benthamiana*, yet it has failed to be as effective in crops because of the developmental pleiotropy and sterility caused by constitutive MR overexpression (Atkins and Voytas, 2020).

Therefore, the research frontier has shifted to regulating the payload to separate regeneration from deleterious growth defects. A more effective category of MRs has been identified by combining Growth-Regulating Factors (GRFs) with their cofactors, GRF-Interacting Factors (GIFs). The resulting chimeras, such as GRF4-GIF1, have shown promising results in overcoming the regeneration bottleneck in difficult-to-regenerate monocots and dicots, such as soybean and sorghum. This approach induces robust cell division without a trade-off in plant reproductive ability (Debernardi et al., 2020). Additionally, this transcriptional reprogramming is increasingly linked to conditioning the cellular environment via small signaling peptides. When co-delivered with MRs, these peptides create a permissive “regenerative niche” that synergizes with GRF-GIF activity to dramatically increase transformation frequencies. Studies have reported the usefulness of peptides such as phytosulfokine (PSK), which reduces the biotic stress response to Agrobacterium infection and physical wounding (Debernardi et al., 2020; Youngstrom et al., 2025; Kowalik et al., 2026).

The operational efficiency of these in planta systems has been further revolutionized by the introduction of visual selection markers to address the challenge of identifying edited shoots against a background of wild-type tissue. In this regard, a prominent study reported integrating the RUBY reporter, a synthetic operon that encodes enzymes for betalain biosynthesis, into the MR-mediated editing cassette, resulting in the visual identification of deep purple-red color in induced meristematic tissues under an optimized protocol for radish (*Raphanus sativus*). This method allows researchers to select gene-edited shoots with the naked eye, avoiding tedious molecular screening or antibiotic selection, which is usually not feasible with soil-grown plants. Lastly, the effectiveness of such “software” updates (genetic regulators) is inherently connected to upgrades in the delivery chassis. Studies emphasize that for the successful delivery of these complex payloads into recalcitrant tissues, designing so-called super-virulent strains of Agrobacterium, which are modified to evade immunity (PTI) caused by pathogen-associated molecular patterns and rely on auxotrophic protection, can be helpful (Goralogia et al., 2025).

In sugar beet, tissue culture remains the only demonstrated route to a transformed plant. Three systems are established: stable Agrobacterium-mediated hypocotyl callus regeneration under kanamycin or hygromycin selection, achieving 1.5–5.1% transformation efficiency and used for the first stable *BvMgCh* and *BvPDS* knockouts (Khan et al., 2026); direct organogenesis from blade-petiole transition-zone explants under G418 selection, though restricted to specific cooperative genotypes (Zhang et al., 2023); and leaf-segment agroinoculation, regenerating transgenic shoots directly from micro-wounded leaf tissue on BAP-supplemented media (Miroshnichenko et al., 2026). Notably, ectopic expression of Arabidopsis *GRF5* (*AtGRF5*) has been demonstrated to circumvent traditional bottlenecks in these *in vitro* systems, enabling the transformation of previously recalcitrant sugar beet varieties and significantly increasing transformation efficiency (Kong et al., 2020; Achary et al., 2026). However, true morphogenic regulator- or peptide-driven *de novo* meristem induction remains an undemonstrated, *in planta* blueprint in the genus *Beta*. Three translational designs have been proposed: a transient *Bvipt*–*BvWUS2* hormone-free regeneration system (Bhattacharjee et al., 2023); geminiviral delivery of *BvBBM*, *BvWUS2*, or *BvGRF5* to the apical meristems of soil-grown plants to generate edited flower buds directly; and *BvREF1* peptide treatment of protoplasts to boost regeneration frequency without stable integration (Gaoua and Arslan, 2026).

### 7.2 Heritable virus-induced genome editing

Although viral vectors have been powerful tools for transient protein expression, their use in creating stable, heritable genome edits has historically been hindered by a biological defense mechanism so potent that it has been difficult to overcome, namely meristem exclusion. To prevent the vertical transmission of pathogens to the germline, plants actively keep most viruses from entering the germline by using RNA interference (RNAi) and other antiviral surveillance pathways (Oh et al., 2021; Shen et al., 2024).

Thus, although traditional Virus-Induced Gene Editing (VIGE) can effectively alter somatic foliar tissue, such edits have traditionally been limited to infected leaves and have rarely been transmitted to seeds. This required a paradoxical return to tissue culture (using infected leaf explants to regenerate plants), thereby negating the major advantage of viral delivery. Recent advancements have fundamentally dismantled this barrier, enabling the production of edited progeny directly from infected parents without somatic regeneration. For instance, researchers have achieved efficient, heritable mutations without tissue culture by customizing a TRV-based virus-induced GE system with tomato-specific promoters and mobile sgRNA elements. This advancement simplifies functional genomics and accelerates breeding in tomatoes and other related crops (Kim et al., 2023; Lee et al., 2025). The most important innovation of Heritable VIGE is the functionalization of the single guide RNA (sgRNA) using endogenous mobility signals. Plants have built-in mechanisms to move certain RNA transcripts, such as those that control flowering or metabolic signaling, into the shoot apical meristem (SAM) to regulate developmental transitions. This mobility relies on the innate mechanism of RNA transfer into the plant shoot apical meristem, which evolved to regulate various physiological processes, including flowering and metabolic signaling (Ellison et al., 2020). Numerous studies have reported fusing sgRNA with the Flowering Locus T (FT) mobile signal or tRNA-like structures. The former, acting as a molecular zip code, helps move the viral-derived CRISPR cargo through plasmodesmata and into the meristematic niche by initiating phloem-mediated transport. The latter pushes the editing complex into germline-precursor cells (the L2 layer of the SAM) that were previously hard to reach (Weiss et al., 2025).

There are reports of successful infection of plants with SAM carrying tobacco ringspot virus (TRSV) and barley yellow striate mosaic virus (BYSMV) to deliver large cargo with the spCas9 effector. In conventional transformation, scientists inoculated the leaves of plants grown in soil. The viral vectors harboring the mobile sgRNA efficiently edited germline cells and produced heritable edited progeny in *N. benthamiana* and wheat, respectively (Yoshida et al., 2024; Qiao et al., 2025). These novel studies lay the foundation for tissue culture-independent heritable GE approaches (Hu et al., 2025).

Diversification of the viral vector toolkit has always been required to expand genome-editing applications to multiple species and to overcome bottlenecks that arise during this expansion beyond model dicots. Among these advancements is the use of an engineered negative-strand RNA virus, Sonchus yellow net virus (SYNV), as an effective carrier of Cas9 editing machinery into *N. benthamiana*, where positive-strand RNA viral vectors (e.g., TRV) are inefficient and unstable. Yet this advancement also has its bottlenecks; among them, a narrow host range is prominent (Ma et al., 2020). Therefore, some reports have suggested engineering other negative-strand RNA viruses with a wider host range, such as tomato spotted wilt virus (TSWV) (Liu et al., 2023). One bottleneck that has remained in VIGE is the cargo capacity of the viral capsids, which have difficulty packaging large enzymes such as SpCas9 (~4 kb).

In sugar beet, viral delivery has been successfully demonstrated systemically, with a BNYVV replicon delivered by Agrobacterium leaf infiltration achieving systemic expression of marker proteins across leaf, stem, and root tissues (Jiang et al., 2019). However, functional gRNA delivery and the resulting editing phenotype remain restricted to somatic tissues, with no reported evidence of germline transmission (Jiang et al., 2019). Concurrently, transient foliar agroinoculation cleaves the invading BCTIV genome at high efficiency, protecting foliage from symptom development (Yıldırım et al., 2023), while heritable gene editing via transmissible viral carriers remains an unachieved milestone. Two designs have been proposed to close this gap: fusing gRNAs to the mobile *BvFT1/BvFT2* transcripts to smuggle a BNYVV-delivered payload into the SAM of vernalized shoots, and single-vector geminiviral replicons that co-express hypercompact TnpB with *BvBBM/BvWUS2* to trigger *de novo* somatic embryogenesis directly *in planta* (Jiang et al., 2019; Weiss et al., 2025; Youngstrom et al., 2025).

### 7.3 Physical and *in planta* delivery systems

Biological reprogramming and viral vectors (though engineered meticulously) have their own limitations. For example, they are restricted to working against certain specific pathogens, and they require detailed information about foreign biological agents. Simultaneously, a wave of physical and mechanical delivery innovations has emerged, and their simplicity and broad genotype-independence rely on innate reproductive and wound-healing pathways, taking advantage of the accessibility of the germline or regenerative tissues.

#### 7.3.1 Cut-Dip-Budding

The Cut-Dip-Buddling (CDB) delivery system is the most disruptive innovation in recent years. The root-suckering ability of a wide range of dicots enables vegetative propagation, and by taking advantage of this feature, CDB can be used to obtain gene-edited plants without the need for sterile conditions, explant preparation, or callus induction (Cao et al., 2023; Hu et al., 2025). The protocol is non-aseptic (**Figure 3**). It involves removing the apical shoot to encourage the growth of adventitious buds (suckers) from the root system. When a wounded root or shoot is dipped into an Agrobacterium suspension, the bacteria infect proliferation-competent cells at the wound site, and the resulting “suckers” develop directly into gene-edited shoots. This method has revolutionized the scope of GE, as it has been successfully employed on recalcitrant crops such as sweet potato (*Ipomoea batatas*) and peanut (*Arachis hypogaea*). Notably, recent advances have made CDB available to succulents and other unique vegetative propagators that were thought to be impossible to edit (Lu et al., 2024; Alam, 2025; Xiao et al., 2025).

CDB works by dipping a wounded root or shoot in an Agrobacterium (or *A. rhizogenes*) suspension carrying the editing construct. The root-inducing (*rol*) genes trigger proliferation of transgenic “hairy roots” at the wound site, which then develop shoot-forming capacity and regenerate edited buds directly in soil (Ma et al., 2020). Validated in rubber root dandelion (*Taraxacum kok-saghyz*) (Li et al., 2026), the method recovers stable mutants in 3–10 weeks versus the 24–40 weeks typical of tissue culture. Translating CDB to sugar beet is limited by the crop’s biology: the taproot is a specialized storage organ with no natural capacity for root-to-shoot conversion or adventitious budding. *A. rhizogenes*-induced hairy roots have been demonstrated in sugar beet to express BNYVV-targeting hairpin dsRNAs for rhizomania resistance but regenerating whole, fertile plants from these hairy roots continues to be a major bottleneck (Pavli et al., 2010). CDB as a whole-plant editing route therefore remains a proposed strategy in the genus Beta; achieving it would likely require co-delivering morphogenic regulators (*BvBBM*, *BvWUS2*) with the editing construct at the wound site to force somatic embryogenesis from injured taproot tissue (Gaoua and Arslan, 2026).

#### 7.3.2 Pollen magnetofection

Chimeras are yet another challenge in somatic cell transformation/editing, as only a fraction of cells are edited while others remain unedited. Haploid germline targeting has emerged as a potential solution for obtaining fully edited progeny. Pollen Magnetofection is an approach that delivers Cas9 editing machinery directly to the zygote using pollen as carriers. The editing machinery is loaded onto magnetic nanoparticles, which are forcibly pulled into pollen grains by applying magnetic fields. These altered pollen grains deliver the machinery to the zygote during fertilization (Babaeianjelodar et al., 2025). This approach yields fully edited seeds in one generation (T1), bypassing the need for somatic regeneration or segregation. The approach is genotype-independent because it takes advantage of the universal pollination process rather than species-specific regeneration pathways (Hu et al., 2025). Cargo is bound to magnetic nanoparticles (e.g., functionalized Fe_3_O_4_) and drawn through the pollen’s germinal apertures under an applied magnetic field, then carried down the pollen tube into the ovule during fertilization. The approach has delivered CRISPR machinery in lily, cotton, pepper, and pumpkin, but a critical reassessment by Vejlupkova et al. (2020) found no reproducible editing across several monocot and dicot species, indicating strong sensitivity to nanoparticle coating and pollen viability (Vejlupkova et al., 2020).

In sugar beet, the method remains a proposed blueprint. Its pollen grains are unusually small (18–22 μm), and penetrating them with a ~160-kDa Cas9 RNP without bursting the tube or killing the sperm cell is a substantial biophysical hurdle. Realizing it would need surface-functionalized nanoparticles (e.g., cell-penetrating peptides) to safely expand the effective pore size, targeted at loci such as the susceptibility genes *BvSGS3*/*BvRDR6* for viral resistance or Rf genes for CMS hybrid seed production.

#### 7.3.3 Grafting mediated editing

This mobile approach involves grafting wild-type shoots (scions) onto transgenic rootstocks that express the Cas9 machinery. The mobile gRNAs, along with the Cas9 effectors, travel through the phloem across the graft junction to the scion flower, leading to the development of edited seeds that germinate into transgene-free progeny containing the desired edits. This approach provides a way to generate transgene-free genome-edited woody perennials where standard transformation remains prohibitive (Yang et al., 2023; Simoni et al., 2025). Researchers established this in Arabidopsis and *Brassica rapa*, grafting wild-type scions onto transgenic Cas9-TLS rootstocks to obtain heritable edits with zero transgene integration in the scion (Yang et al., 2023). The approach is limited to dicots, since monocots lack the vascular cambium needed for a graft union.

Sugar beet, as a dicot, does have a vascular cambium, but its shoot apical meristem sits on a compressed crown directly above the taproot with no elongated stem for conventional grafting, making the technique anatomically awkward rather than impossible. Micro-grafting onto Cas9-expressing rootstocks, such as those already validated with the pKSE401-*PDS* or pKSE401-*MgCh* visual-marker constructs (Khan et al., 2026), to translocate mobile gRNAs into elite commercial scions remains a proposed strategy, one that would bypass both callus-phase regeneration and the 2-year vernalization wait for transgene segregation.

### 7.4 Automation, high-throughput systems, and biofoundries

Although biological and physical innovations address the possibility of transformation in recalcitrant species, they do not necessarily address scalability. Traditional plant transformation is a highly artisanal, labor-intensive process that relies heavily on researchers’ skills and experience. This reliance on extensive labor limits scalability and, consequently, the throughput needed for commercial breeding and functional genomics. In recent years, there has been a major shift toward industrializing this process through automation, incorporating robots into a Biofoundry. A mechanism describing how Biofoundry can be useful in automated plant screening is shown in **Figure 3**.

The manual bottleneck of preparing and infecting vectors has begun to be automated through the integration of open-source laboratory robotics. Annese et al. (2025) reported a semi-automated workflow that employs the Opentrons OT-2 platform to standardize Agrobacterium-mediated transformation (Annese et al., 2025). While working on the transformation of the model system Marchantia polymorpha, the researchers observed similar editing efficiencies in both manual and automated systems, yet the reproducibility was significantly enhanced by the later approach of incorporating programmed liquid-handling robots to perform the freeze-thaw, plating, and resuspension steps needed in transformation. This change eliminates the risk of human error and contamination, and hundreds of constructs can be processed at the same time. This standardization is required to conduct Design-Build-Test-Learn (DBTL) cycles in synthetic biology in plants, in which the rate of DNA assembly has far exceeded the rate of phenotypic validation (Annese et al., 2025; Brandizzi et al., 2025).

The biological protocol should be simplified so it can be manipulated by robots and make the most of automation. Minimal Tissue Culture Transformation (MTCT) and Tissue Culture-Free Transformation (TCFT) have been reported as two different models of high-throughput engineering (Zhong et al., 2025). In contrast to traditional protocols, which involve several manual transfers between the induction, selection, and rooting media-steps and are not easily automated, MTCT systems simplify the process to a single infection-and-incubation step, followed by direct transfer to soil or automated image-based screening.

Genotype independence is another feature these automated systems offer, eliminating the need to curate ratios of different growth hormones and their combinations while simultaneously minimizing the plant immune response against hypervirulent strains of Agrobacterium (Goralogia et al., 2025). Robotic handling, MTCT protocols, scalability, and automation, combined with the use of visual selection markers like RUBY, are driving the rapid growth of Plant Biofoundries. This combined approach aims to minimize the extensive labor required to acquire or develop successful, desirable edits through sensitive and complex editing machinery, as they offer laser-based explant cutting, robotic Agrobacterium infection, and robust automated phenotyping with hyperspectral imaging. This shift from petri dish to field and from robot to field marks the coming of age of plant genome engineering as an experimental methodology, moving it into a manufacturing field (Indibi et al., 2025). Genomic resources and scalable workflows are paving the way for automated genome engineering in sugar beet. High-throughput protoplast screening is still an emerging field, but standardized validation workflows are being employed to uncover key trait loci, including viral resistance factors (Rollwage et al., 2024). Tissue-culture scalability is being enhanced by the use of semi-automated temporary immersion bioreactors that speed callus maturation, to avoid the traditional solid-agar bottlenecks. Current roadmaps involve the integration of high-throughput field phenotyping using multispectral computer vision (Nguyen et al., 2025)with automated biofoundry workflows to systematically accelerate breeding for complex agronomic traits such as drought resilience in the genus *Beta*.

### 7.5 The complexity gap

Despite these developments, there remains a serious Complexity Gap. While the simple knockouts (NHEJ) or base edits have been developed with the advent of TCI, there is still much work to be done to bring complex editing systems, such as PE, or large DNA insertions (HDR), under the TCI umbrella. This complexity gap is particularly large in recalcitrant, highly heterozygous dicots such as sugar beet because of structural, genetic, and physiological limitations (Mukherjee and Gantait, 2023). The genome of sugar beet is highly repetitive, with about 60%–70% of the genome being heterochromatin, which is largely composed of various LTR retrotransposons (e.g., the *Beetle7, Cotzilla*, and *Elbe3–2* families) that limit chromatin accessibility and editing efficiency (Zakrzewski et al., 2017). In addition, sugar beet has a strict biennial growth cycle and is completely self-incompatible, leading to high inbreeding depression and the loss of elite heterozygous parental backgrounds if breeders depend on multi-generational meiotic segregation to eliminate transgenic backbones (Dohm et al., 2014; Mukherjee and Gantait, 2023).

Although simpler TCI has been able to bypass tissue culture in some species, including Kalanchoe with 63%–74% stable editing and potato with up to 28% stable integration, their ability to provide multi-component, macromolecular editing architectures is severely limited (Ma et al., 2025). The narrow size-exclusion limit of the rigid plant cell wall (~5–20 nm) and the thick epicuticular leaf cuticle are biophysical delivery barriers that remain to be solved for delivery of bulky complexes (Demirer et al., 2019). Therefore, although transient, high throughput sugar beet protoplast assays are commonly used to assess the kinetics of sgRNA cutting on endogenous targets, the recovery of whole, non-chimeric regenerants without soma clonal variation or soma clonal-associated DNA methylation shifts is a significant challenge (Zakrzewski et al., 2017; Rollwage et al., 2024).

This is because the frequency of HDR in somatic cells is low and the size of viral vectors is limited, meaning that even genome writing (insertion of >10 kilobase metabolic pathways) is still dependent on conventional regeneration. In fact, PE is a very versatile SSB method for introducing precise point mutations or small insertions without DSBs, but its efficiency is very unstable and species-dependent (Tian et al., 2025; Zhang et al., 2026). The PE efficiency varies widely in plant protoplast assays and is strongly target-site and species-specific, with a marked decrease in recalcitrant dicotyledonous crops compared to monocots. To increase these insertion limits, researchers have developed large-scale genome writers such as PrimeRoot, which can insert up to 11.1 kb, and transposable element systems such as TATSI, which can insert up to 8.6 kb with an 18.2% efficiency in crops (Sun et al., 2024; Muzaffar et al., 2026; Zhang et al., 2026). Additionally, novel recombinase platforms-such as Kp03-enable highly efficient, site-specific integration of kilobase-length DNA fragments in plant cells (Yan et al., 2025). The packaging of these large multi-component cargos (e.g., nCas9-RT-recombinase complexes with multiple engineered pegRNAs), however, is much larger than the cargo packaging capacity of conventional transient delivery vehicles such as deconstructed TRV vectors.

The translation of advanced tools such as PrimeRoot into a TCI-compatible format is still a challenge to fully realize the potential of precision crop engineering. To overcome this complexity gap, researchers have suggested using deep learning frameworks such as DeepCRISPR to computationally predict on-target efficiency and design optimized, PAM-relaxed Cas9 variants (Chuai et al., 2018; Walton et al., 2020; Hina et al., 2026). These reduced, hypercompact editing systems are proposed to be delivered to intact sugar beet meristems through non-integrating polymer-functionalized SWCNTs as a critical future avenue to achieve stable, transgene-free, large-fragment trait stacking, including the repression of competing vacuolar invertase genes to prevent post-harvest sucrose loss, directly in elite commercial clonal germplasms (Demirer et al., 2019; Mukherjee and Gantait, 2023).

## 8 Biotic resilience via insect precision engineering

Insects provide important ecosystem services in the plant biosphere, such as pollination and pest control, but also have the capacity to vector various pathogens and cause diseases (Fiallo-Olivé et al., 2020; Li et al., 2024). The transition into the post-Cas9 era is defined by a fundamental shift toward cross-kingdom precision engineering, where the advanced molecular approaches used to edit plant genomes are being deployed to reprogram insect vectors for holistic crop protection. Scientists have used chemical and biological methods to tackle these issues. However, unscientific and non-standard application of insecticides has led to pest resistance, which impedes pest control effectiveness (Alyokhin and Chen, 2017; Moyes et al., 2017). The evolution of insecticide resistance in insects has been a major challenge in controlling insects. For instance, increased expression of genes such as *Glutathione S-transferases* (GSTs) and *CYP6F1* in *Anopheles gambiae* and *Culex pipiens pallens* confers resistance to DDT and deltamethrin (Roberts et al., 1991; Gong et al., 2022). Mutations in genes alter the amino acid sequence at target sites, resulting in diminished insecticide efficacy and resistance in insects (Muthusamy and Shivakumar, 2015). However, by aligning the discovery of novel resistance alleles in insects with the sophisticated search-and-replace capabilities of tools like Prime Editing, researchers can now design dual-front defense strategies.

### 8.1 Molecular decoders

CRISPR/Cas technology has transitioned from a tool for simple gene disruption into a high-resolution molecular decoder used to identify and validate candidates for next-generation search-and-replace modifications. In major agricultural pests, particularly those in the orders Lepidoptera and Hemiptera, CRISPR/Cas9 has enabled the functional validation of genes involved in detoxification and toxin reception, identifying specific alleles for precise replacement.

For instance, knocking out cadherin (*HaCAD* gene), a receptor for the Bt toxin Cry1A in Helicoverpa armigera, makes insects 49 times more resistant to Cry1Ac (Wang et al., 2016). CRISPR/Cas9-based deletion of *HaRdl-1* and *HaRdl-2*, which encode critical subunits of insect γ-aminobutyric acid (GABA) receptors in H. armigera, altered the insect’s sensitivity to cyclodiene and phenylpyrazole (Wang et al., 2020). In insects such as *Plutella xylostella*, CRISPR/Cas9-based double and quadruple gene knockouts showed functional redundancy among *ABCC2*, *ABCC3*, *APN1*, and *APN3a* and their involvement in resistance to the Bt Cry1Ac toxin (Sun et al., 2022). Researchers demonstrated functional redundancy among *ABCC2*, *ABCC3*, and *PxmALP*, which act as Bt Cry1 toxin receptors (Sun et al., 2025). Sun et al. (2025) further identified non-receptor paralogs of these genes that can help reduce the fitness costs of Cry1Ac resistance (Sun et al., 2025). A study showed the role of the methionine aminopeptidase gene *PxMetAP1* in regulating Bt Cry1Ac tolerance. Using CRISPR/Cas9, they demonstrated that disrupting *PxMetAP1* significantly increased the insect’s susceptibility to Cry1Ac, indicating that this gene contributes to Bt tolerance (Ye et al., 2022).

In *Plutella xylostella* and *Tuta absoluta*, two-point mutations, *F1845Y* and *V1848I*, in the *IVS6* segment of the voltage-gated sodium channel contribute to indoxacarb resistance. This was further validated by Samantsidis et al. using CRISPR/Cas9-mediated GE in *Drosophila melanogaster*. Bioassays revealed that homozygous *F1845Y* or *V1848I* mutants had moderate resistance to indoxacarb, while the *F1845Y* homozygous mutant exhibited significant resistance to metaflumizone (Samantsidis et al., 2019).

In *Spodoptera exigua*, mutation of the *nicotinic acetylcholine receptor α6* subunit (*Seα6*-KO) is linked to resistance to spinosad and spinetoram (Zuo et al., 2022). Furthermore, researchers used a reverse-genetics approach to validate the functional role of the *G275E* mutation in the *nAChR α6* subunit of *S. exigua* in spinosyn resistance (Zuo et al., 2022). In a study by Shi et al., CRISPR/Cas9-mediated knockout of the CYP9A gene cluster was correlated with high levels of pyrethroid resistance (Shi et al., 2023). Strong resistance to Bt Cry toxins, especially Cry1A and Cry1F, in *Ostrinia furnacalis* is linked to mutations in an ABC transporter gene, further validated by a CRISPR-mediated 8-bp deletion in the *ABCC2* gene (Wang et al., 2020). In *Nilaparvata lugens*, P450-mediated metabolic detoxification, particularly *CYP6ER1* overexpression, is an important resistance mechanism to neonicotinoid insecticides. A study showed that a CRISPR-based CYP6ER1 knockout increased sensitivity to imidacloprid and thiacloprid (Huang et al., 2021). In *Myzus persicae*, a single point mutation at position 81 (R81T) in the *nAChR β1* subunit increases tolerance to neonicotinoid insecticides. CRISPR/Cas9-mediated *R81T* substitution in *D. melanogaster* showed enhanced resistance to neonicotinoids and a significant decline in overall fitness (Homem et al., 2020).

Sugar beet is heavily affected by foliar pests like the beet armyworm (*S. exigua*) and root-feeding pests like the sugar beet root maggot (*Tetanops myopaeformis*) (Mukherjee and Gantait, 2023). Although commercial sugar beet protection historically relied on transgenic expression of insecticidal Bt Cry toxins (such as Cry1Ab) to control armyworm damage (Jafari et al., 2009), modern researchers have proposed using CRISPR-mediated promoter editing of endogenous flavonoid and phenolic biosynthetic pathways to induce local biosynthesis of defense metabolites directly at the site of insect feeding (Gurel et al., 2008; Mukherjee and Gantait, 2023). Furthermore, target genes identified from insect-side decoder screens provide a molecular blueprint for developing targeted HIGS sprays or seed coatings that selectively disrupt essential larval transcripts without compromising the genetic integrity of the elite heterozygous crop background (Cui et al., 2017).

Importantly, these insect-side discoveries are being integrated with plant-side immunity engineering. For example, CRISPR/Cas9 GE technology developed *gmcdpk38* mutants of *Glycine max* with enhanced resistance to common cutworm (CCW) (Li et al., 2022). In rice, resistance to pests like stem borers and plant hoppers was enhanced by blocking serotonin production (a neurotransmitter produced in response to insect infestation) through CRISPR/Cas9 genetic modification (Lu et al., 2018). By enhancing expression of genes encoding the biosynthesis of secondary metabolites using CRISPR/Cas9, insect herbivory in plants can also be reduced, as in maize (Kumari et al., 2024). CRISPR/Cas9-mediated flavonoid biosynthesis in soybean induced a mutation in the *GmUGT* gene and enhanced resistance to leaf-chewing insects (Zhang et al., 2022).

### 8.2 ReMOT control

The shift toward the post-cas9 era and TCI paradigm intends to minimize labor-intensive bottlenecks of *in vitro* manipulation. However, this is not confined to the plant kingdom as there has been reported a breakthrough known as “ReMOT Control” (Receptor-Mediated Ovary Transduction of Cargo) has emerged to dismantle the traditional requirement for embryo microinjection. It is the insect equivalent of plant callus induction. In this process the Cas9 is delivered into vitellogenic adult females using an ovary-targeting peptide (BtKV). This approach enables efficient, heritable GE in *Bemisia tabaci* through simple adult injections to produce edited progeny without sterile culture (Heu et al., 2020). This is one-step editing approach, where the physical barrier of the cell (plant cell wall or insect embryo) is overcome through biochemical recruitment rather than mechanical force.

For sugar beet, where whitefly vectors act as devastating carriers for viral pathogens like Beet curly top virus (BPYV) (Seethapathy et al., 2025), deploying ReMOT control represents a highly innovative proposed strategy to suppress vector transmission directly in the field (Cui et al., 2017). This biochemically recruited, injection-based editing bypasses the physical barriers of both the plant cell wall and the delicate insect embryo, facilitating a dual-front approach that couples targeted host plant resistance with the evolutionary suppression of vector populations.

### 8.3 Gene drives and active inheritance control

The post-Cas9 era is defined by a shift from stochastic Mendelian inheritance toward Ecosystem-Scale Writing, where precise modifications are propagated across the crop biosphere. In entomology, the CRISPR-based gene drives act as molecular writers, ensuring that desired traits bypass standard segregation to achieve rapid pest management. CRISPR-based gene drives can be used to knock out specific target genes in insect populations, resulting in reduced survival, altered sex ratios, or impaired fertility. In addition, editing plant immunity genes, such as susceptibility genes (S-genes), resistance genes (R-genes), or genes regulating plant-insect interactions, offers the potential to enhance host resistance against insect pests. As a case in point, CRISPR/Cas9-based mutation of the abdominal-A (*Pxabd-A*) gene in *P. xylostella* resulted in lethality and severe abdominal defects (Huang et al., 2016).

In the context of the sugar beet agricultural ecosystem, executing these gene drive designs is proposed as a landscape-scale management tool to control the beet armyworm. By coupling CRISPR-based homing gene drives with lethal developmental mutations (such as inducing male sterility), researchers propose to actively drive population decline or sex-ratio distortion across open-field populations (Cui et al., 2017). Simultaneously, editing endogenous plant immunity loci offers a complementary, host-directed mechanism to deter larval herbivory (Rollwage et al., 2024). Several studies on enhancing resistance against insects are shown in **Table 4**. Thus, by moving from disruptive molecular scissors to the sophisticated modulators, the post-Cas9 toolkit enables the mutual design of both plant resilience and insect population dynamics.

**Table 4:** Representative CRISPR/Cas applications for elucidating pest control resistance mechanisms in insects.

Order	Species	Target gene	Gene function	CRISPR modification	Phenotype observed	Reference
*Lepidoptera*	*Helicoverpa armigera*	*HaABCA2*	ATP-binding cassette transporter, Receptor for Bt Cry2 toxin	Two knockout strains	Resistance to Cry2Ab	(Wang et al., 2017)
		*HaABCC2*	Receptor for Bt Cry1Ac toxin	Monogenic knockout	512-fold resistance to Cry1Ac	(Zhang et al., 2021)
		*HaCad + HaABCC2*	Dual Cry1Ac receptor pathways	Double knockout	6273-fold resistance to Cry1Ac	(Zhang et al., 2021)
		*CYP6AE P450* cluster	Detoxification	Cluster knockout	Altered sensitivity to esfenvalerate and indoxacarb	(Wang et al., 2018)
		*GST* gene cluster	Detoxification	Cluster knockout	Enhanced susceptibility to lambda-cyhalothrin	(Wang et al., 2018)
	*Helicoverpa zea*	*HzABCA2*	Receptor for Bt Cry2Ab	Monogenic knockout	Resistance to Cry2Ab toxin	(Fabrick et al., 2022)
	*Pectinophora. gossypiella*	*PgABCA2*	Receptor for Bt Cry2Ab	Monogenic knockout	Resistance to Cry2Ab toxin	(Fabrick et al., 2021)
	*Trichoplusia ni*	*ABCC2*	Receptor for Bt Cry1Ac toxin	Monogenic knockout	Resistance to Cry1Ac	(Ma et al., 2022)
	*Tuta absoluta*	*RyR*	Target of diamide insecticides	Monogenic Knock in	Resistance to diamide insecticides	(Douris et al., 2017)
	*Spodoptera frugiperda*	ABCC2	ABC transporter receptor for Bt toxins	Knockout	Conferred high resistance to Bt Cry1F toxins without altering chemical pesticide susceptibility.	(Abdelgaffar et al., 2021)
*Hemiptera*	*Bemisia tabaci*	*ACC*	Target of tetronic/tetramic acid insecticides	Monogenic knockout	Resistance to spiromesifen and spirotetramat	(Lueke et al., 2020)
	*Nilaparvata lugens*	*CYP6ER1*	Mediates resistance to neonicotinoids	Monogenic knockout	Insecticide resistance	(Zhang et al., 2023)

## 9 Conclusion, future perspective and role toward sustainable agriculture

The identification of GE with CRISPR/Cas has set a solid foundation for modern biotechnology. Automation of these genome-editing systems will aid in optimizing them, especially for crops. New plant-breeding techniques and the design of constructs with synthetic biology will enable researchers to concentrate on editing platforms for specific applications, such as screening, long mutations and on/off switches for promoters. Their efficiency and stability will be improved in the future by research efforts. The main bottleneck to accelerating the development of edited crops is the need for tissue-culture-independent techniques. Tissue-culture-independent approaches will help speed up crop improvement programs. Screening for resistance genes and targeting them to control plant pathogens while minimizing impact on the ecosystem is also required. This will also be possible in the future with the help of synthetic biology and automated screening systems. The ultimate success of these advanced technologies for sugar beet relies heavily on overcoming the inherent tissue culture recalcitrance of sugar beet. The future of CRISPR in *Beta vulgaris* is promising, with the potential to revolutionize the crop’s development by combining emerging in planta delivery systems and morphogenic regulators with precise editors, thereby eliminating transgenic footprints and ensuring the crop’s climate-resilient and disease-resistant future.

The post-Cas9 era of plant synthetic biology is ushered in by hypercompact, PAM-relaxed nucleases, including prokaryotic TnpB and eukaryotic Fanzors. These miniaturized effectors can be used to successfully edit intact SAMs when delivered via transient, tissue-culture-independent vectors such as deconstructed viral replicon systems or single-walled carbon nanotubes. At the same time, advanced genome writers such as PrimeRoot and site-specific recombinases (e.g., Kp03) enable kilobase-scale trait stacking without DSBs in crop plants. In sugar beet, the use of these macromolecular tools provides a very promising proposed roadmap for stacking complex agronomic traits, such as salt-tolerant transcription factor networks and sucrose-partitioning transporters (BvTST2;1), directly into elite heterozygous lines without disrupting the desirable parental genetic background.

These editing strategies are becoming more and more successful, and are now supported by the incorporation of artificial intelligence and deep-learning prediction models (e.g., DeepCRISPR) for guide RNA design and chromatin accessibility models. High resolution contiguous genome assemblies such as EL10 can be used as a predictive in silico template to systematically target AT-rich promoters and avoid dense repetitive heterochromatic regions dominated by retrotransposons. Finally, the complex global regulatory environment is a major hurdle to commercialization, and a coordinated international transition from the traditional transgenic (SDN3) regulatory framework to SDN1 and SDN2 is essential. Modern biotechnology can sustainably shift the genus Beta from a traditional sucrose source to a highly productive biomanufacturing platform for industrial and pharmaceutical applications by using transient, DNA-free delivery vehicles and precise multi-genic writers.
